# Insights from Integrative Systematics Reveal Cryptic Diversity in *Pristimantis* Frogs (Anura: Craugastoridae) from the Upper Amazon Basin

**DOI:** 10.1371/journal.pone.0143392

**Published:** 2015-11-24

**Authors:** H. Mauricio Ortega-Andrade, Octavio R. Rojas-Soto, Jorge H. Valencia, Alejandro Espinosa de los Monteros, Juan J. Morrone, Santiago R. Ron, David C. Cannatella

**Affiliations:** 1 Laboratorio de Biogeografía, Red de Biología Evolutiva, Instituto de Ecología A.C., Xalapa, Veracruz, México; 2 Sección de Vertebrados, División de Herpetología, Museo Ecuatoriano de Ciencias Naturales, Quito, Ecuador; 3 Fundación EcoCiencia, Programa para la Conservación de Especies y Ecosistemas Amenazados en Ecuador, Quito, Ecuador; 4 Fundación Herpetológica Gustavo Orcés, Quito, Ecuador; 5 Red de Biología Evolutiva, Instituto de Ecología A.C., Xalapa, Veracruz, México; 6 Departamento de Biología Evolutiva, Museo de Zoología ‘Alfonso L. Herrera’, Facultad de Ciencias, Universidad Nacional Autónoma de México (UNAM), México City, México; 7 Museo de Zoología, Departamento de Ciencias Biológicas, Pontificia Universidad Católica del Ecuador, Quito, Ecuador; 8 Department of Integrative Biology and Biodiversity Collections, The University of Texas, Austin, Texas, United States of America; BiK-F Biodiversity and Climate Research Center, GERMANY

## Abstract

Pluralistic approaches to taxonomy facilitate a more complete appraisal of biodiversity, especially the diversification of cryptic species. Although species delimitation has traditionally been based primarily on morphological differences, the integration of new methods allows diverse lines of evidence to solve the problem. Robber frogs (*Pristimantis*) are exemplary, as many of the species show high morphological variation within populations, but few traits that are diagnostic of species. We used a combination of DNA sequences from three mitochondrial genes, morphometric data, and comparisons of ecological niche models (ENMs) to infer a phylogenetic hypothesis for the *Pristimantis acuminatus* complex. Molecular phylogenetic analyses revealed a close relationship between three new species—*Pristimantis enigmaticus* sp. nov., *P*. *limoncochensis* sp. nov. and *P*. *omeviridis* sp. nov.—originally confused with *Pristimantis acuminatus*. In combination with morphometric data and geographic distributions, several morphological characters such as degree of tympanum exposure, skin texture, ulnar/tarsal tubercles and sexual secondary characters (vocal slits and nuptial pads in males) were found to be useful for diagnosing species in the complex. Multivariate discriminant analyses provided a successful classification rate for 83–100% of specimens. Discriminant analysis of localities in environmental niche space showed a successful classification rate of 75–98%. Identity tests of ENMs rejected hypotheses of niche equivalency, although not strongly because the high values on niche overlap. *Pristimantis acuminatus* and *P*. *enigmaticus* sp. nov. are distributed along the lowlands of central–southern Ecuador and northern Peru, in contrast with *P*. *limoncochensis* sp. nov. and *P*. *omeviridis* sp. nov., which are found in northern Ecuador and southern Colombia, up to 1200 m in the upper Amazon Basin. The methods used herein provide an integrated framework for inventorying the greatly underestimated biodiversity in Amazonia.

## Introduction

The practice of species delimitation has been widely discussed [1,2] because of its central importance to biodiversity [3–5]. The combined use of techniques (e.g. niche modeling, morphometric analyses, and phylogeography) to evaluate distributional, phenotypic, and genetic variation in populations [6–9], has promoted a paradigm shift in the practice of species delimitation [10–12], one which has immediate consequences for conservation biology, biogeography and evolutionary biology [13–16].

Information on species diversity is particularly important for regions such as the upper Amazon Basin in the western lowlands of Colombia, Ecuador and Peru, which holds the richest, most diverse, and complex amphibian assemblages from any area on Earth [17–23]. Amazonia is also an extremely threatened ecosystem, with about 1.8 million ha/year of primary forest lost since 1980, resulting in fragmentation of continuous forest into islands [24,25]. Knowledge of the ecology, biogeography and diversification patterns of herpetological assemblages in this threatened core of mega-diversity remains poor [26,27], despite characterization of the Amazon Basin as one of the best studied regions in South America [22].

Frogs of the genus *Pristimantis* (Terrarana: Craugastoridae) comprise one of the most striking, richest and understudied groups in the Neotropics [11,28,29]. Similar to other Terrarana, *Pristimantis* have direct development (no tadpole phase), which is associated with terrestrial habitats [30]. With nearly 469 species (~7% of amphibians worldwide) distributed mainly in South America [31,32], this group is considered highly threatened, with at least 35% of the species included in the Red List [33]. Most of the threatened species show a marked endemism in the tropical Andes, and are considered sensitive to environmental disturbances and habitat loss [34–36]. Species delimitation is particularly difficult within young evolutionary radiations such as *Pristimantis* [11,37–40]. For example, Pinto-Sánchez et al. [41] demonstrated that most of phenetic taxonomic species of Central American *Pristimantis* are not monophyletic

The Canelos Robber Frog, *Pristimantis acuminatus* Shreve [42], is a widely distributed species in Amazonia [30]. Curiously, as noted in the original description ([42] page 217), the holotype of *P*. *acuminatus* lacks a distinct tympanic annulus, whereas the annulus is distinct in the two paratypes. Since then, the acuminate snout shape and the absence of the tympanum have been used commonly as diagnostic characters to assign similar greenish *Pristimantis* to *P*. *acuminatus* [43–45]. However, it is now suspected that *P*. *acuminatus* is a complex of species [30].

To resolve this taxonomic problem, we reviewed the type specimens of *P*. *acuminatus* as well as a broad sample of specimens from field and museum collections. We herein describe three new species based on an integrative approach that incorporates phylogenetic, morphometric and ecological data.

## Materials and Methods

### Ethics statement

Voucher specimens and tissue samples were obtained following ethical and technical protocols [46]. Vouchers were euthanized with lidocaine hydrochloride 2%, fixed in 10% buffered formalin and then later transferred to 70% ethanol. Liver and thigh muscle were preserved in 95% ethanol for DNA extraction. Euthanasia protocols and research permits to work in the Amazon Basin of Ecuador were approved by Gabriela Montoya of the Ministerio del Ambiente del Ecuador (authorization No. 001-12-IC-FAU-DNB/MA and No. 001-IC-FAU/FLO-DRFN-P/MA). Voucher specimens and tissue samples were deposited at Museo de Zoología, Pontificia Universidad Católica del Ecuador (QCAZ).

### Protocol for species delimitation

We evaluated the status of populations in the *Pristimantis acuminatus* complex as distinct independent evolutionary lineages, under the general framework of the unified species concept as outlined by de Queiroz [47]. We followed the consensus protocol for integrative taxonomy proposed by Padial *et al*. [48]. Our modified protocol includes several steps: 1) a reduction of taxon sampling for groups of specimens (e.g. based on differences in a single morphological character), 2) comparative molecular analyses, 3) a second step reduction of taxon sampling (e.g. qualitative morphological differences) congruent with the phylogenetic analyses, and 4) comparative analyses of various lines of evidence for delimiting species (e.g. morphometric, ecological, biogeographical, etc.). Specimens for morphological analyses and ecological modeling were selected after having the results of phylogenetic analyses, in the step two.

### Focal species and genetic sampling

A total of 33 specimens of the *Pristimantis acuminatus* complex and *P*. *tantanti* (an Amazonian species similar to *P*. *acuminatus* [44]) were included in the phylogenetic analysis. The outgroup species included *Craugastor longirostris* and *Oreobates cruralis*, as representatives of sister clades of *Pristimantis*, and *Pristimantis crucifer* as a distantly related species within the genus. *Craugastor longirostris* was used to root the resulting phylogenetic trees. Localities, sample numbers, coordinates and GenBank accession numbers for all samples are provided in S1 Table. Field work was carried out by the senior author at Limoncocha (Sucumbíos province, March 2012 and May 2013) and Tukupi (Morona Santiago province, May 2012), Ecuador (S2 Table). Specimens were collected during night surveys, from 18h00−00h00 at the end of the rainy season (February through April), using headlamps to find individuals on vegetation. We measured each individual with a digital caliper (0.05 mm accuracy) and photographed them with a digital camera (Canon Rebel T2). We followed the technique of Visual Encounter Surveys (VES) [49]. Color photographs and notes on color, ecology, latitude/longitude/elevation (using a GPS Garmin^®^ Montana 650) were taken in the field for each specimen.

We examined the type-series of *Pristimantis acuminatus* (holotype MCZ A19951, paratypes MCZ A19949–50), *P*. *pseudoacuminatus* (holotype MCZ A19948), *P*. *tantanti* (holotype MHNSM 23942), and 135 specimens loaned from the following institutions (S2 Table): Colombia: Instituto de Ciencias Naturales, Universidad Nacional de Colombia, Bogotá (ICN); Instituto Alexander von Humboldt, Bogotá (IAvH). Ecuador: Fundación Herpetológica Gustavo Orcés, Quito (FHGO); Museo Ecuatoriano de Ciencias Naturales, Quito (DHMECN); Museo de Zoología–Pontificia Universidad Católica del Ecuador, Quito (QCAZ). Peru: Museo de Historia Natural Javier Prado de Lima (MHNJP); Centro de Ornitología y Biodiversidad, Lima (CORBIDI); Museo de Historia Natural de la Universidad Nacional Mayor de San Marcos, Lima (MHNSM); Museo de Historia Natural Universidad Nacional de San Antonio Abad, Cusco (MHNC), Giussepe Gagliardi’s collection at Instituto de Investigaciones de la Amazonía Peruana, Iquitos (GGU-IIAP). USA: American Museum of Natural History, New York, USA (AMNH); Museum of Comparative Zoology, Harvard University (MCZ); National Museum of Natural History, Washington, D.C. (USNM), and Natural History Museum, The University of Kansas (KU). Each locality from museums databases was carefully reviewed (lat–long coordinates) to correct imprecise geo-references in decimal degrees, based on the WGS 84 datum.

### DNA amplification

DNA was extracted from most tissue samples using a single-step method with acid guanidinium thiocyanate [50] or by using a UltraClean^®^ Tissue & Cells DNA Isolation Kit (MO-BIO Laboratories, Inc., Carlsbad, CA, USA), following the manufacturer’s manual. Three mitochondrial genes– 16S rRNA (16S), 12S rRNA (12S), and the Folmer Region or ‘‘Barcode of Life” fragment of the Cytochrome Oxidase sub-unit I (COI; [51]) gene–were amplified (S3 Table). Polymerase chain reaction was carried out under locus-specific optimal annealing temperatures following protocols detailed by Pinto-Sánchez et al. [41]. PCR products were cleaned using the UltraClean PCR Clean-Up Kit (MO-BIO Laboratories, Inc., Carlsbad, CA, USA) or by Exo I/SAP digest, and sequenced in both directions by Macrogen Co. Ltd. (South Korea). Sequences were edited and aligned in GENEIOUS v5.4.7 (Biomatters, Auckland, New Zealand). Multiple sequence alignments were generated using MAFFT v7.017 [52] with default gap opening cost and other settings configured in GENEIOUS. Leading and trailing ends were trimmed manually to remove any missing data. To identify related sequences, a Nucleotide Blast search was carried out using the NCBI database (http://blast.ncbi.nlm.nih.gov/Blast.cgi).

### Phylogenetic analyses

Because our combined data set comprised two ribosomal genes with secondary structure (12S and 16S) and one protein-coding mitochondrial gene (COI), application of a single nucleotide substitution model was unlikely to provide a particularly good fit to the data [53]. Partitions were defined *a priori* and Bayes factors were used to choose among alternative partitioning strategies. Three distinct partitioning strategies were evaluated: 1) one partition (three genes concatenated), 2) three partitions, by gene (12S, 16S, and COI), and 3) five partitions (12S, 16S, and COI further partitioned by codon position). Bayes factors were calculated using twice the difference in the marginal model likelihoods [2ln(B_10_)] as estimated from the harmonic mean of the likelihoods of the posterior sample of trees for the simplest model (M_0_) against the more complex model (M_1_) [53,54]. A Bayes factor greater than 10 was considered as very strong support for the more complex model [55]. For each partitioning scheme, the matrix included the same number of terminals and characters.

Phylogenetic analyses were conducted using Maximum Likelihood (ML) and Bayesian Methods (BM) on individual genes and on concatenated datasets. Prior to ML and BM analyses, we used JModeltest 2.3.1 [56] through the Phylemon 2.0 Server [57] to select the optimal model for each gene and codon position for COI (Table 1). Due to the small sample size in our matrix among genes and partitions (12S = 13 sequences; 16S = 28 sequences; COI = 27 sequences), we used a corrected Akaike Information Criterion (AICc) to select the best-fitting model [58].

**Table 1:** Summary of taxon sampling and best-fitting models for combined and individual genes, taxa, and characters.

Gene/Codon position	Taxa	Number of characters	Best-fitting model	AIC corrected value	– ln Likelihood	I	G	C	V	PI	S
All data	36	1997	GTR+I+G	15043	7438	0.32	0.50	1280	699	367	325
12S	16	763	GTR+G	5446	2682	n/d	0.37	473	280	121	153
16S	31	564	GTR+G	3952	1897	n/d	0.23	389	175	90	84
COI	28	670	HKY+I	5374	2620	0.59	n/d	418	244	156	88
COI, 1st		223	TrNef+I	1393	616	0.69	n/d	177	44	24	20
COI, 2nd		223	F81	862	350	n/d	n/d	211	9	4	5
COI, 3rt		224	HKY	2874	1355	n/d	n/d	30	191	128	63

Evolution models were evaluated by comparisons of the Akaike Information Criteria (AICc). I = Proportion of invariable sites; G = Gamma distribution; C = Conserved sites; V = Variable sites; PI = Parsimony-Informative sites; S = Singleton sites. Outgroup species correspond to *Craugastor longirostris*, *Oreobates cruralis* and *Pristimantis crucifer*.

ML was carried out in GARLI v2.0 (Genetic Algorithm for Rapid Likelihood Inference; [59]) through the CIPRES portal (http://www.molecularevolution.org/index). We ran 5 independent searches, whereas support for the nodes were calculated by a search using 100 bootstrap replicates. Tree searches were performed with stepwise-addition starting trees (streefname = stepwise), 5000000 generations as maximum for each run (stopgen = 5000000), saving every 100 generations (saveevery = 100), a run termination threshold of 20000 generations without topology improvement (genthreshfortopoterm = 20000), and a termination threshold value of 0.01 in the increase in lnL required for any new topology (significanttopochange = 0.01); other parameters were used with the default setting [59]. Mesquite [60] was used to generate a majority-rule consensus tree from the bootstrap replicates. The Bayesian phylogenetic analysess were implemented in MrBayes v3.2.2 [60] in the CIPRES portal [61] (http://www.molecularevolution.org). The search consisted of two parallel runs for 10 million generations each. Three heated chains (heating parameter = 0.2) were used for each run Trees and their associated parameters were sampled every 1000 generations. Convergence of the two runs was judged sufficient using 0.008 as the cut-off for the average deviation of the split frequencies. The sampled log-likelihood values were visualized using Tracer v1.6 [62], and adequate mixing of the chain was assessed using an effective sample size (ESS) >200 and a Potential Scale Reduction Factor (PSRF) value near 1.0 as criteria [63]. The first 25% of generations were discarded as burn-in. Gene tree concordance was assessed by analyzing each locus individually using Bayesian analysis.

To estimate species-trees, we used the coalescent model in *BEAST 1.8.2 [64] using a yule prior for tree topology [65,66]. To allow for differences in the molecular characteristics of each sequence, the substitution and clock models parameters were set as unlinked for COI, 12S, and 16S. We estimated genetic distances between species for each gene using the uncorrected p-distance in MEGA 6 [67]. Pinto-Sánchez *et al*. [41] suggested an estimated root for this complex in about 4.16 Myr old. We used this date to infer specific nucleotide substitution rates for each locus (i.e. 0.004 Myr^−1^ for the 12S; 0.0025 Myr^−1^ for 16S; and 0.0097 Myr^–1^ for COI) to estimate lineage divergence (time to most recent common ancestor, TMRCA). These mutation rates were set as priors for the ucld.mean parameter in the *BEAST analyses. We performed two independent runs of 120 million generations with a 10% burnin to reach reliable ESS values (>200). We use TRACER v1.6. [66] to assess stationarity and convergence of runs.

### Species delimitation

We used a Poison tree processes (PTP) model for species delimitation [68] to infer the most likely species number in our data, as implemented in bPTP server (http://species.h-its.org/ptp/). This method has commonly used to explore putative species boundaries using only nucleotide substitution on a given phylogenetic tree, implementing a model assuming gene tree branch lengths generated by two independent Poisson process classes (within- and among-species substitution events). PTP is a single-locus species delimitation method that outperforms the commonly used Generalized Mixed Yule Coalescent (GMYC), without requiring an ultrametric tree [68,69]. As input, we used a makimum likelihood best solution tree of concatenated dataset, estimated by GARLI. We ran the PTP analysis using 100,000 MCMC generations, with a thinning value of 100, a burn-in of 0.1, and opted for removing the outgroup to improve species delimitation. Convergence of MCMC chain was confirmed visually as recommended [68].

### Morphological analyses

We selected specimens for morphological analysis after a second step reduction of taxon sampling, based on qualitative morphological differences, congruent with the phylogenetic analyses (see details in the Integrative Working Protocol, S1 Table). We used the characters, terminology and format of Duellman and Lehr [30]. Measurements were taken from the right side of specimens, and, if this was not measurable, from the left side. Sex was determined by direct inspection of gonads. Measurements were taken on 14 morphometric characters with the aid of dial calipers (~0.1 mm precision): (1) snout–vent length (SVL) = distance from tip snout to posterior margin of vent; (2) head width (HW) = greatest width of head at level of jaw articulation; (3) head length (HL) = distance from the tip of snout to posterior angle of jaw articulation; (4) horizontal eye diameter (ED) = distance between anterior and posterior margin (corner) of eye; (5) Interorbital distance (IOD) = the width of the braincase between the orbits; (6) eye-nostril distance (EN) = distance from posterior margin of nostril to anterior margin of eye; (7) width of upper eyelid (EW) = horizontal length of the upper eyelid; (8) tympanum diameter (TD) = distance between external anterior and posterior margins of tympanic annulus (not used for multivariate analyses because tympanum is absent in *P*. *acuminatus sensu lato*); (9) femur length (FL) = length of femur from vent to knee; (10) tibia length (TL) = length of flexed leg from knee to heel; (11) foot length (FtL) = distance from heel to tip of toe IV, including in the measurement the length of tarsus and foot; (12) hand length (HdL) = distance from proximal border of thenar tubercle to tip of Finger III; (13) disc diameter of Finger III (F3) = horizontal width of the disc of Finger III; and (14) disc diameter of Toe IV(T4) = horizontal width of the disc in Toe IV.

We conducted multivariate analyses with morphological measurements to reduce morphometric variables (principal component analysis, PCA) and to delimitate species (discriminant analysis). We performed normed principal component analyses as implemented in the *ade4* R package [70]. We evaluated the effect of variables on percent explained variance for males and females separately. Additionally, we performed a discriminant analysis (DA) to identify morphometric traits that contribute most to species separation. Specimens used in DA were defined following the phylogenetic analyses. To avoid size-dependent correlation effects, regression residuals on log-transformed data were calculated using snout-vent length (SVL) as variable. We applied a forward stepwise procedure (tolerance = 1.0), with residuals and the natural logarithm of SVL as variables, to evaluate whether species were separated in morphological space and which morphometric characters contribute to the separation. A matrix of squared Mahalanobis distances was used to compare differences between species and to classify cases assigned by the DA.

Finally, we applied the Kruskal-Wallis non-parametric test (KW-test, corrected with exact Monte Carlo Test, 10000 samples and 95% confidence intervals) to compare overall morphological variation among species. We preferred this test over a parametric test (e.g. MANOVA), due to the nature of the data (e.g. unbalanced data, small sample size, lack of normality). For variables with significant p-values from the KW test, we applied non-parametric Mann-Whitney pairwise comparisons. All analyses and statistics were developed in PASW Statistics v18.0 (WinWrap Basic).

### Environmental species delimitation by ecological niche models (ENMs)

Similar to morphological analysis, after a second step reduction of taxon sampling, we selected collection localities for ecological analysis congruent with the phylogeny (see details in Integrative Working Protocol). The ecological niche models (ENMs) were developed under the assumption that organisms have distinct ecological requirements that determine their occurrences in time and space [71]. It has been argued the ENMs are useful for delimiting cryptic species [8,72], but this is controversial. We collected georeferenced data from 130 specimens known from 93 unique localities available in herpetological collections (S2 Table).

We conducted multivariate analyses with 19 environmental variables from WorldClim project [73]. These parameters incorporate annual trends (e.g. mean annual temperature, annual precipitation), aspects of seasonality (e.g. annual range in temperature and precipitation) and extreme or potentially limiting environmental factors (e.g. temperature of the coldest and warmest months, and precipitation of the wettest and driest months). We performed a normed Principal Component Analyses (PCA-env) and Discriminant Analyses (DA-env) to define environmental traits that are most informative for distinguishing the ENMs of each species. The localities used in DA-env were defined based on clades recovered in the phylogenetic analyses. A matrix of squared Mahalanobis distances was used to compare differences among species and determine the number of cases correctly and incorrectly assigned by DA-env.

We also modeled habitat suitability for population of a species within the *Pristimantis acuminatus* complex using MaxEnt Software version 3.3.3a [74,75]. MaxEnt estimates the probability of distribution that has maximum entropy by applying the following principle: the expected value for each feature (e.g. climatic variables) must equal the empirical average value for points relating to known presence. The algorithm performs a certain number of iterations until reaching a convergence limit. The final map represents a favorability rating ranging from 0 (unsuitable) to 1 (perfectly adequate) [75]. The program uses two input resources: localities of the species record (presence-only data) and digital layers of the environmental conditions of a given area. The set of localities was randomly partitioned for each species, in 70% as training data and 30% for testing the model (see below concerning ROC curve criteria to validating data). The environmental variables that were relevant to each model’s reconstruction are reported based on the multivariate analyses and results of the jackknife test calculated by MaxEnt [76]. This allowed us to reduce over-fitting of the distribution models generated for each species [7,77]. Resolution grid cell size, or pixel size, was 0.0083 degrees, which corresponds to ~1 km^2^ in each raster.

The overall predictive distribution models for each species were generated with 5000 iterations of the complete training dataset. The analysis was done without "clamping" or "extrapolation" to avoid unsupported extrapolations on the extremes of the ecological variables. All other parameters of MaxEnt were maintained as default settings. To aid model validation and interpretation, it is usually desirable to distinguish suitable from unsuitable areas by setting a decision threshold above which model output is considered to be a prediction of the species presence. There is no rule for setting these thresholds because their values depend on the data used or the purpose of the map, which will vary from species to species [78]. Because we used all training datasets to generate validated ENMs, we decided to apply the Minimum Training Presence (MTP) value as the threshold to convert the probabilistic values (logistic ranges from 0 to 1) into a binary presence-absence map. The occurrence extension range was created from a convex hull polygon derived from the union of all points from verified localities. Using a polygon might underestimate the distribution of the species, especially when additional localities of occurrence are expected to be found. Nonetheless, we applied this method because it is commonly used to evaluate and compare the extension range for threatened species [79,80]. Spatial analyses and map algebra were done using ArcMap 10 Software; the convex hull polygon was calculated from Minimum Bounding Geometry routine in ArcTool Box [81].

The performance of MaxEnt models are usually evaluated using a ROC curve (Receiver Operating Characteristic curve; [75]), a statistical technique that has become a dominant tool in evaluating species distribution model [82]. However, several problems have been associated with this technique [83,84]; one is that the two error components (omission and commission) are inappropriately weighted equally. Accordingly, we use partial-area ROC, which evaluates only over the spectrum of the prediction and allows for differential weighting of the two error components [13,84]. The Area Under the ROC Curve (AUC) was limited to the proportional areas over which models actually made predictions and only omission errors of <5% were considered [84]. We calculated partial AUCs with the Tool for Partial-ROC V. 1.0. [85] using 30% of the original data for independent model evaluation. We present the partial ROC results as the ratio of the model AUC to the null expectation (AUC ratio; [84]). Bootstrapping analyses of AUCs were done by resampling (with replacement) 50% of the points 1000 times from the overall pool of data. One-tailed significance of differences in AUC (e.g. deviation from the line of null expectation) was assessed via fitting a standard normal variate (z-score) and calculating the probability that the mean AUC ratio is ≤1 [84].

### Ecological equivalency of niche models

Schoener’s D metrics [86,87] were calculated from ENMs for each pair of species using ENMtools software [6]. It has been argued that the ecological interpretation of this index suggest that the suitability scores generated by MaxEnt are relative proportional to species abundance [6]; thus we prefer this metric over the similarity statistic I-index [87]. The D-index measures the overlap or similarity of the suitability area predicted by MaxEnt for pairs of *species* (considering the logistic probabilities of pixels) and represents the proportion of niche coincidence between them. We applied a randomization test proposed by Warren *et al*. [87], the Identity Test, to explore whether pairs of species´ models were more different than would be expected given the underlying environmental differences between the areas in which they occur. This test assumes that probabilities of ENMs produced by two populations are identical (= equivalent) if sampling is unbiased with respect to the species environmental tolerances [6]. The hypothesis of niche identity is rejected when the observed D-value is significantly lower than the values expected from the pseudoreplicate data [6,87]. The significance of differences in Schoener’s D metric from the null expectation (one-tailed) was assessed by counting the number of bootstrap replicates with lower values than the observed D-index.

## Results

### Phylogenetics and species delimitation

**Fig 1:**
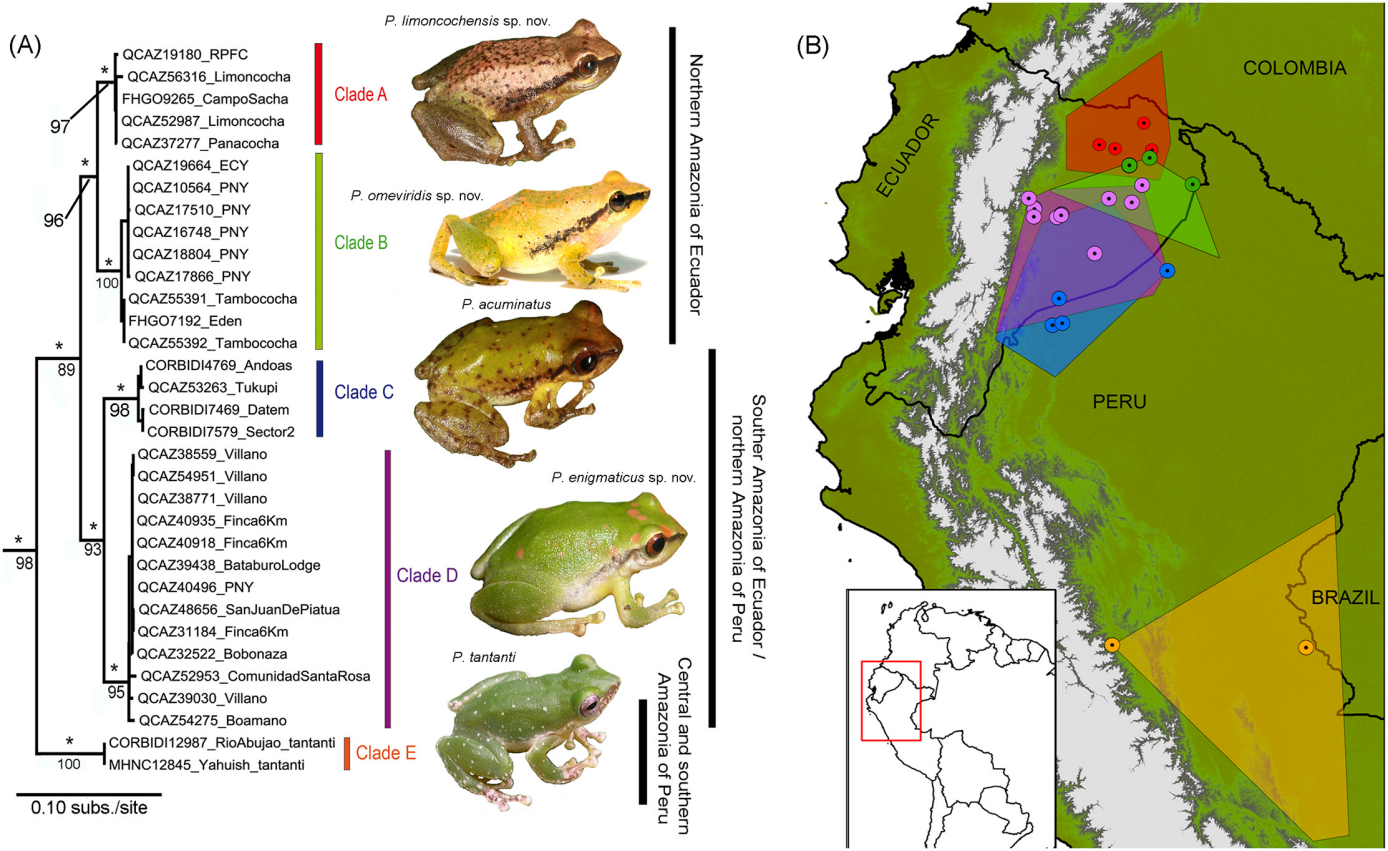
Phylogeny and distribution of the *Pristimantis acuminatus* group in the Amazon Basin. **(A)** Optimal maximum likelihood tree (log likelihood = -6762.95) inferred from a partitioned analysis of 1997 aligned sites of the 12S, 16S and COI (by codon position) mtDNA genes, showing the phylogenetic relationships among 33 specimens identified as *P*. *acuminatus sensu lato* and *P*. *tantanti* from the Amazon basin. Clade A = *Pristimantis limoncochensis* sp. nov., clade B = *P*. *omeviridis* sp. nov., clade C = *P*. *acuminatus sensu stricto*, clade D = *P*. *enigmaticus* sp. nov., and clade E = *P*. *tantanti*. Stars denote clades with Bayesian posterior probability values1; numbers below clades represent non-parametric bootstrap support values. **(B)** Areas of distribution for species in the complex. Dotted circles = Localities of collection from specimens used for the phylogenetic analyses; Polygons = occurrence areas drawn as minimum convex polygons for each clade based on specimens reviewed in collections (S2 Table). Colors of clades in the phylogenetic tree correspond to colors of polygons and dotted circles on the map.

The genetic sampling corresponds to 33 specimens (12S: 13 sequences, 16S: 27 sequences, COI: 27 sequences; see S1 Table) identified as *P*. *acuminatus sensu lato* and *P*. *tantanti* from the Amazon basin of Ecuador and Peru. The parameter estimates for the best-fit models for each mitochondrial gene are summarized in Table 1. The best topology (log likelihood = -6762.95, ML analysis) was obtained from a 5-partitioned matrix of combined mtDNA (S4 Table), with 1997 characters, under a GTR+G (12S, 16S) and TrNef+I, F81, and HKY substitution models for the first, second, and third positions of COI. The phylogeny derived from concatenated sequences shows remarkably high divergences in mitochondrial genes (Fig 1, S1 Fig), among populations of “*P*. *acuminatus*”, with four distinct and well-supported clades (Bayesian posterior probabilities, pp = 1.0; non-parametric bootstrap, bs = 89–100). Uncorrected p-distances between species are detailed in Table 2. Distances ranged from 1.8–4.7% (3.3±1.5 standard error) for 12S, 1.6–3.8% (2.8±0.8 SE) for 16S, and 4.7–8.7% (7.3±1.5 SE) for COI. Distances between the four clades and their sister taxon, *Pristimantis tantanti*, ranges from 5.3–11.5% (7.6±3.4% SE). The PTP model for species delimitation identified four putative species within what is known as *Pristimantis acuminatus* (S1D Fig), with highly support values (0.91–0.99).

**Table 2:** Congruence of key diagnostic characters for morphology, biogeography and genetic distances between species resolved by the phylogeny (12S + 16S + COI).

Character	*P*. *limoncochensis* (Clade A)	*P*. *omeviridis* (Clade B)	*P*. *acuminatus* (Clade C)	*P*. *enigmaticus* (Clade D)
Skin texture	Smooth	Smooth	Shagreen	Shagreen
Tympanic annulus beneath skin	Absent	Present	Absent	Present
Males with vocal slits and nuptial pads	Absent	Absent	Present	Absent
Tarsal fold or tubercles	Small tubercles	Large tubercles	Smooth	Small tarsal fold
Distribution in Amazonia	Northern Ecuador and southern Colombia	Northern Ecuador and southern Colombia	Central and southern Ecuador; northern Peru	Central and southern Ecuador; northern Peru
Elevational range (average±sd)	199–593 m (275.8±75.6)	154–382 m (251.4±52.8)	175–1123 m (413.2±269.2)	169–956 m (501.3±287)
Latitudinal range	N1.1442°–S0.91082°	S0.55689°–S2.14833°	S1.21°–S4.04308°	S1.19972°–S3.34207°
12S p-distance (%)	1.8–3.3% (2.6±1.1)	1.8–4.7% (3.3±2.1)	3.3–4.7% (4.0±1.0)	No data
16S p-distance (%)	1.6–3.0% (2.46±0.8)	1.6–3.8%(2.96±1.2)	2.1–3.8%(2.96±0.9)	2.1–3.5%(2.80±0.7)
COI p-distance (%)	4.7–8.7% (7.3±2.2)	4.7–7.9% (6.7±1.8)	6.3–8.7% (7.5±1.2)	6.3–8.5% (7.6±1.1)

The number of base substitutions per site from averaging over all sequence pairs between species is shown as an uncorrected p-distance value (%) by gene.

**Fig 2:**
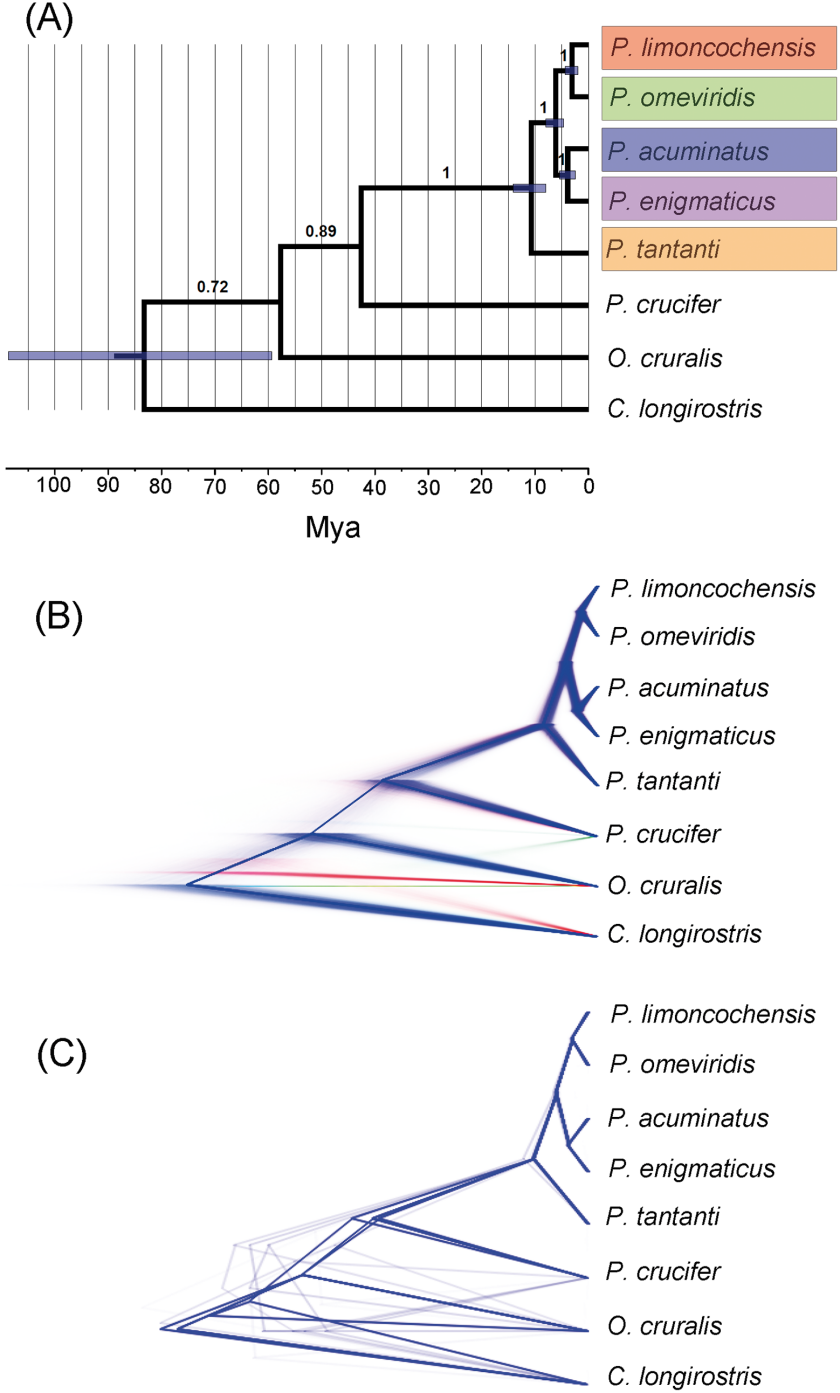
Coalescent species reconstruction in *BEAST. **(A)** Species tree chronogram with posterior probabilities, **(B)** DensiTree visualization of all estimated gene trees, and **(C)** DensiTree visualization of possible consensus trees for the even loci. Colors of lineages recovered by the species-tree **(A)** correspond to colors of polygons and doted circles in the geography of Fig 1B.

The *BEAST analysis recovered the same topology estimated by the phylogenetic analyses (2a). We found congruence among individual mitochondrial trees, recovering a sister-group relationship between *Pristimantis limoncochensis* sp. nov. (clade A) and *P*. *omeviridis* sp. nov. (clade B), and a sister-group relationship for *P*. *acuminatus* (clade C) + *P*. *enigmaticus* sp. nov. (clade D), all of them with high support (pp = 1.0). All gene-tree reconstructions and consensus trees (Fig 2b and 2c) show a strong support for the monophyly of the complex, with a TMRCA for the entire ingroup estimated to be 10.86 Myr (8.01–14.1 95% HPD). The divergence time between northern and southern populations in the Amazonia of Ecuador is estimated to be 6.21 Myr (4.65–8.02 95% HPD). The TMRCA of *Pristimantis limoncochensis* sp. nov. and *P*. *omeviridis* sp. nov. is estimated to be 3.15 Myr (1.94–4.39 95% HPD), whereas the time of divergence between *P*. *acuminatus* and *P*. *enigmaticus* sp. nov. is estimated to be 3.91 Myr (2.41–5.41 Myr 95% HPD).

The most relevant results are: (i) The phylogeny recovered four divergent lineages, supported by the PTP model, for specimens identified as *P*. *acuminatus* in the upper Amazon Basin, and (ii) the phylogenetic tree shows that populations located in the northern portion of the Amazon basin of Ecuador and southern Colombia group in clades A and B which are sister to populations in the southern part of Ecuador and northern Peru (clades C and D).

### Morphometric species delimitation

**Fig 3:**
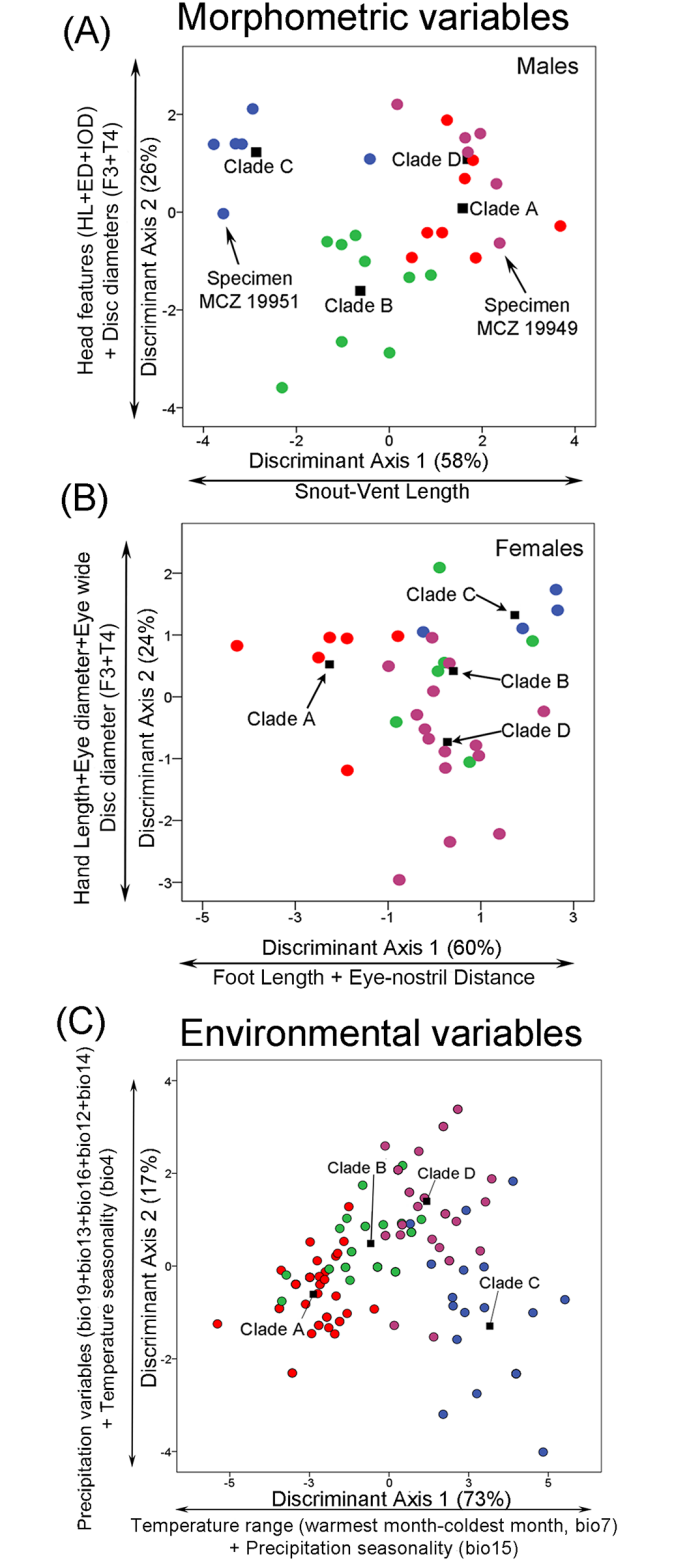
Discriminant analyses of morphological and environmental variables. Projections of morphometrics for **(A)** males, **(B)** females and **(C)** environmental datasets by discriminant analyses for the *Pristimantis acuminatus* complex. Dots represents specimens in A-B and localities in C. Colors represents the clades recovered by the phylogenetic analysis (Fig 1), whereas the black squares represent their centroids. Note that the holotype (MCZ A19951) and paratype (MCZ A19949) of *Pristimantis acuminatus* are assigned to different species (Clades C and D, respectively) by the discriminant analysis **(A)**. The 93% of the males and 81% of the females were morphological successfully assigned to species, whereas the 88% of the localities were successfully classified in the environmental space.

A total of 62 adult specimens from the *Pristimantis acuminatus* complex were examined for morphometric analyses. Data used for principal component and discriminant analyses are provided in the S5 Table. A summary of the descriptive statistics for morphometric measurements of males and females of the *P*. *acuminatus* complex is presented in Table 3. Loadings, eigenvalues and percentage of variance explained by Principal Components and Functions in DA are provided in S5 and S6 Tables. Projections of morphometric variables in morphological space are represented in Fig 3.

**Table 3:** Descriptive morphometric statistics for species of the *Pristimantis acuminatus* complex.

Variable	Species				Kruskal-Wallis Test (KW)	
	*P*. *limoncochensis* Clade A (*n* = 8)	*P*. *omeviridis* Clade B (*n* = 9)	*P*. *acuminatus* Clade C (*n* = 6)	*P*. *enigmaticus* Clade D (*n* = 6)	KW	*P-value* (Monte Carlo)
Males						
SVL	20.70±1.07	20.80±1.77	22.80±1.12	20.70±2.32	6.87	0.07
	18.67–22.18	17.77–23.31	20.91–24.01	18.51–24.79		
HL	7.60±0.47	7.80±0.93	8.10±0.17	7.40±0.72	4.25	0.24
	6.73–8.15	6.86–9.73	7.80–8.29	6.92–8.58		
HW	7.30±0.56	7.30±0.62	8.10±0.45	7.00±0.60	8.27*	0.03
	6.42–8.16	6.25–8.04	7.39–8.50	6.48–7.98		
EN	2.40±0.25	2.40±0.19	2.70±0.14	2.60±0.17	8.88*	0.02
	2.16–2.83	2.11–2.60	2.54–2.90	2.34–2.84		
ED	2.70±0.25	2.80±0.30	2.70±0.21	2.70±0.35	1.71	0.65
	2.19–2.94	2.27–3.20	2.51–3.10	2.30–3.31		
IOD	3.20±0.43	2.90±0.35	3.40±0.45	3.10±0.25	6.26	0.10
	2.84–4.15	2.17–3.30	2.98–4.20	2.84–3.50		
EW	1.90±0.23	1.90±0.25	2.00±0.24	1.90±0.32	1.30	0.74
	1.60–2.20	1.57–2.30	1.67–2.36	1.68–2.53		
TD^a^	–	1.00±0.22	–	1.00±0.12	10.00^b^	0.37
	–	0.66–1.30	–	0.84–1.08		
FL	10.00±0.68	10.10±0.76	10.80±0.48	10.10±0.72	6.04	0.10
	8.96–10.73	8.46–10.77	9.85–11.12	9.14–11.31		
TL	10.80±0.59	10.50±0.97	11.30±0.96	10.60±0.81	3.21	0.37
	9.98–11.81	8.38–11.57	9.90–12.87	9.73–12.12		
FtL	14.20±0.85	13.50±1.17	14.90±0.99	13.90±1.30	5.55	0.13
	12.60–15.17	10.88–15.11	13.47–16.32	12.33–16.19		
HdL	5.70±0.47	5.80±0.63	6.30±0.40	6.00±0.58	4.61	0.20
	4.80–6.24	4.71–6.49	5.85–6.96	5.44–7.04		
F3	1.10±0.15	1.00±0.20	1.30±0.12	1.10±0.20	6.58	0.08
	0.91–1.30	0.65–1.30	1.06–1.40	0.92–1.49		
T4	1.00±0.10	1.00±0.19	1.20±0.20	1.10±0.21	4.44	0.22
	0.91–1.22	0.64–1.30	0.99–1.43	0.87–1.48		
Females						
SVL	29.00±1.25	28.6±1.92	30.50±2.96	30.80±2.92	3.40	0.34
	27.73–30.79	26.10–30.91	27.10–33.45	26.38–36.37		
HL	10.40±1.05	9.70±0.63	11.20±2.59	10.70±1.04	4.60	0.21
	9.45–12.43	8.60–10.39	9.10–14.94	9.10–12.50		
HW	9.80±0.41	9.40±0.72	10.90±1.87	10.50±0.86	8.28*	0.03
	9.36–10.38	8.10–10.14	8.80–12.8	8.80–12.18		
EN	3.00±0.30	3.10±0.19	3.40±0.31	3.30±0.33	6.30	0.09
	2.63–3.48	2.90–3.40	3.10–3.78	2.77–3.90		
ED	3.30±0.24	3.20±0.18	3.40±0.23	3.40±0.37	1.50	0.71
	2.99–3.58	2.90–3.42	3.10–3.64	2.72–4.00		
IOD	4.30±0.08	3.80±0.20	4.30±0.73	4.20±0.39	8.07*	0.03
	4.21–4.44	3.57–4.10	3.30–4.96	3.74–4.88		
EW	2.30±0.36	2.40±0.13	2.60±0.36	2.40±0.23	2.70	0.49
	1.89–2.96	2.20–2.60	2.10–2.89	2.04–2.90		
TD^a^	–	1.20±0.18	–	1.40±0.24	22.00^b^	0.08
	–	0.95–1.41	–	0.92–1.90		
FL	13.80±0.77	13.60±0.49	14.40±1.70	14.40±1.04	4.40	0.23
	12.65–14.87	12.95–14.40	11.90–15.58	12.30–15.89		
TL	14.10±0.50	14.20±0.33	15.50±2.19	15.10±0.9	7.89*	0.04
	13.52–14.88	13.70–14.60	12.80–17.94	13.40–16.42		
FtL	18.50±1.15	19.00±0.88	20.40±2.69	20.50±1.49	8.83*	0.02
	16.61–19.49	17.75–20.30	16.60–22.36	17.21–22.47		
HdL	8.00±0.34	7.90±0.64	8.40±1.35	8.70±0.71	7.90*	0.04
	7.64–8.61	7.20–8.70	6.40–9.44	7.20–9.78		
F3	1.50±0.21	1.60±0.22	1.90±0.52	1.80±0.29	6.20	0.09
	1.28–1.80	1.30–1.86	1.10–2.28	1.16–2.10		
T4	1.60±0.27	1.60±0.18	1.60±0.45	1.70±0.28	2.40	0.50
	1.16–1.91	1.39–1.84	1.10–2.08	1.13–2.10		

Mean ± SD are given with range below. Abbreviations are: SVL = snout–vent length; HL = head width; HW = head length; ED = horizontal eye diameter; IOD = Interorbital distance; EN = eye-nostril distance; EW = width of upper eyelid; TD = tympanic diameter (not used for PCA); FL = femur length; TL = tibia length; FtL = foot length; HdL = hand length; F3 = disc diameter on finger III; and T4 = disc diameter on toe IV. All measurements are in mm.

^a^ Tympanum diameter was not used in the PCA (see Materials and Methods),

^b^ but a Mann-Whitney paired test was used to compare tympanum size between species.

*Variables with significant statistics at P = 0.05.

The normed PCA shows limited evidence of grouping. A slight separation of male specimens between clade A and clade C can be in males (S2 Fig). Variables involved in such separation are body size (snout-vent length) and Foot+Tibia length (S6 Table). Species can be successfully separated based on quantitative traits by means of a discriminant function (Fig 3 and S7 Table). In males, 93% of specimens were correctly assigned to each species; body size (snout-vent length), head traits (head length, eye diameter, and inter-orbital distance) and disc diameter (Finger III and Toe IV) were the variables that explained 84% of the classification by the functions 1 and 2 (Fig 3 and Table 4). In females, the classification value falls to 81% success of correctly assigning the specimens to the species (Table 4). Variables involved in discrimination of the females were mainly related to foot length, hand length, head traits (eye-nostril distance, eye diameter, and eyelid width), with 84% of variance explained by the functions 1 and 2 (Fig 3). A Kruskal-Wallis analysis between morphometric variables revealed significant differences among species within the *Pristimantis acuminatus* complex (Table 3).

**Table 4:** Results of successful classification in morphological-space (males/females) and environmental-space (localities), with percentage in parentheses, assigned to each clade by discriminant analysis.

	Males (93%)				Females (81%)				Localities (88%)			
Species (Clade)	A	B	C	D	A	B	C	D	A	B	C	D
*P*. *limoncochensis* (A)	7 (88%)	-	-	1 (13%)	5 (83%)	-	-	1 (17%)	42 (98%)	1 (2%)	-	-
*P*. *omeviridis* (B)	1 (11%)	8 (89%)	-	-	-	5 (83%)	-	1 (17%)	2 (6%)	27 (87%)	-	2 (6%)
*P*. *acuminatus* (C)	-	-	6 (100%)	-	-	-	3 (75%)	1 (25%)	-	1 (4%)	23 (88%	2 (8%)
*P*. *enigmaticus* (D)	-	-	-	6 (100%)	1 (7%)	1 (7%)	1 (7%)	12 (80%)	-	5 (16%)	3 (9%)	24 (75%)

The numbers in the cells indicate the number of individuals, followed by the percentage in parentheses. Overall percentages of successful classification are given in parentheses for males, females and localities.

### Environmental species delimitation

The phylogenetic analysis placed 28 localities in clade A, 24 in clade B, 18 in clade C and 23 in clade D, all in the Upper Amazon Basin of Ecuador and Peru. A summary of environmental variables, loadings, eigenvalues and percentage of variance explained by principal components (PCA-env) and functions in discriminant analysis (DA-env) is presented in S8 Table. Projections of climatic variables in environmental space are presented in Fig 3. The normed PCA-env shows limited evidence to separate localities by each species group, in spite of high correlation with axis 1 (96% of variance explained; S2 Fig). Annual Rainfall, Precipitation of Wettest Quarter, Precipitation of Warmest Quarter and Precipitation of Coldest Quarter are contribute to the two most important principal components. In contrast to PCA-env, the DA-env discriminates species shown a better classification in a context of discriminant function (Fig 3), 90% of localities were correctly assigned to each species (Table 4). The first two axes of the DA-env analysis explain nearly 90% of overall variance (Fig 3) based on three temperature variables (Mean Diurnal Temperature Range, Seasonality, and Temperature Range) and six precipitation variables (Annual Precipitation, Precipitation in the Wettest Month, Precipitation in the Driest Month, Precipitation Seasonality, Precipitation in the Driest Quarter, and Precipitation of Coldest Quarter).

### Ecological niche models

**Fig 4:**
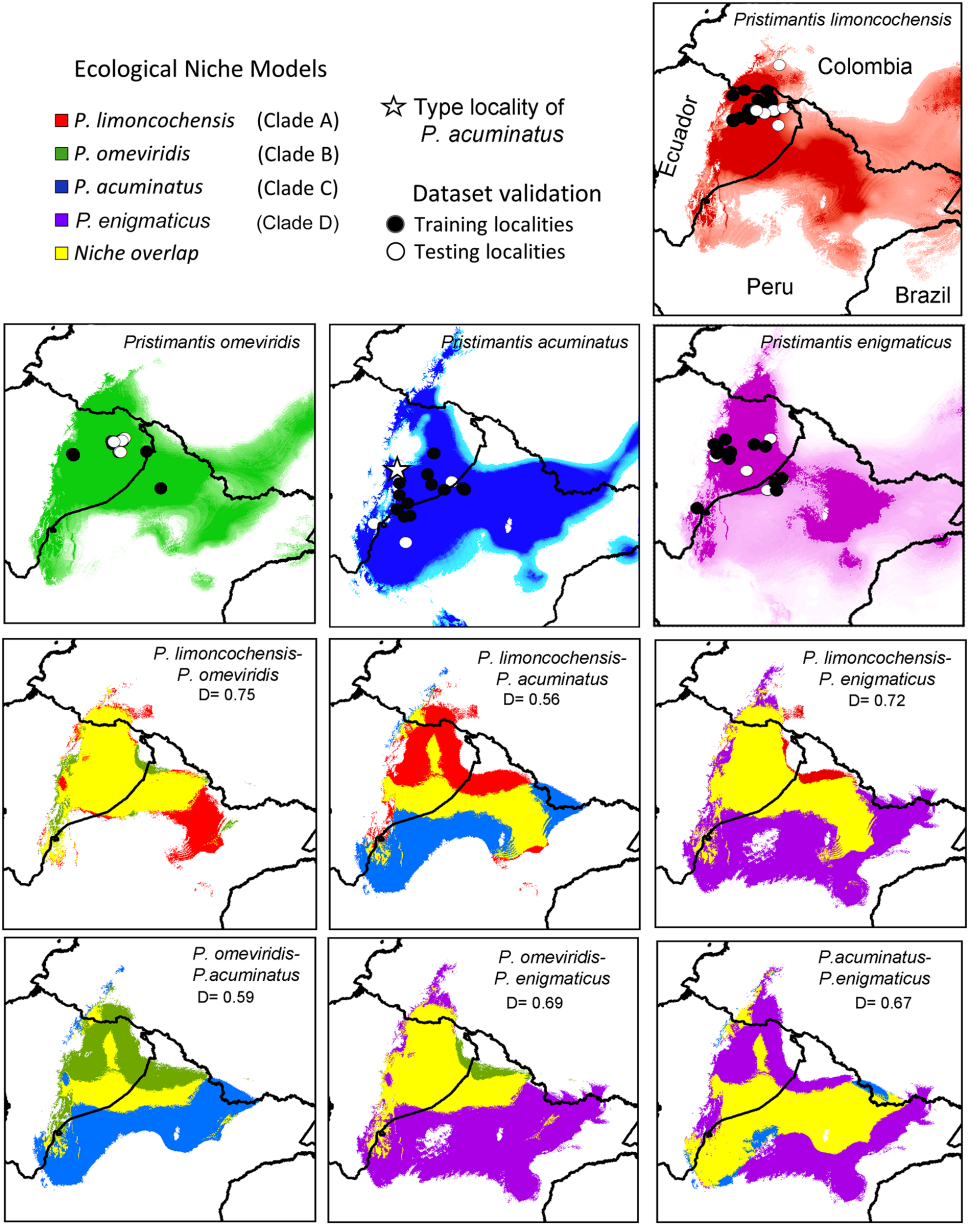
Ecological niche models (ENM) and niche overlap between species within the *Pristimantis acuminatus* complex. The yellow regions indicate niche overlap in pairwise comparisons of each species; D = values of Schoener's D index for niche overlap. The Minimum Training Presence (MTP) threshold and a Partial-ROC dataset was used to validate the models; over-predicted areas in the extreme west Amazonia are not shown. The star represents the type-locality of *Pristimantis acuminatus* (MCZ 19951) at Canelos, Pastaza Province, Ecuador.

Descriptive statistics and spatial representation of the ecological niche models are shown in Fig 4 and Table 5, respectively. Models were constructed with 21 points for clade A (7 for testing), 18 points for clade B (6 for testing), 13 points for clade C (5 for testing), and 17 points for clade D (6 for testing). The AUC-ratios for the ENMs range from 1.9±0.053 (clade A) to 1.99±0.007 (clade B). Each AUC-ratio is statistically differeny from the null model AUC ratio of 1.0 (*z-*test, *P*<<0.01). Variables with the most important contribution to models were Precipitation of the Driest Month (Bio14) for clades A, B and D and Precipitation Seasonality (BIO15) for clade C (S3 Fig). The ecological niche model generated for clade D covers the widest area (~370,019 km^2^), whereas the model for clade B includes the most restricted area (~147,787 km^2^). The ercentage of non-overlapping or exclusive areas for each model ranges from 2%, in clade A to 27% in clade D (Table 5).

**Table 5:** Descriptive statistics for ecological niche models (ENMs) and their spatial representations for species within the *Pristimantis acuminatus* complex.

Species (Clade)	Logistic value for MTP threshold	AUC ratio	Predicted model (km^2^)	Overall Non-overlapped area (km^2^)	Overall Overlapped area (km^2^)^+^	Occurrence area (km^2^)
*P*. *limoncochensis* (A)	0.140	1.9+0.053*	177,042 (100%)	3,259 (2%)	173,783 (98%)	28,140 (16%)
*P*. *omeviridis* (B)	0.343	1.99+0.007*	147,787 (100%)	4,661 (3%)	143,126 (97%)	22,950 (16%)
*P*. *acuminatus* (C)	0.414	1.93+0.022*	201,174 (100%)	14,437 (7%)	186,737 (93%)	50,951 (25%)
*P*. *enigmaticus* (D)	0.090	1.95+0.021*	370,019 (100%)	103,359 (28%)	266,660 (72%)	53,203 (14%)

Minimum training presence (MTP) was used as the threshold to define the suitability areas in the models, whereas the occurrence area was estimated with a convex hull polygon.

* indicates that the z-score is statistically significant at P<<0.01 compared to null model (AUC ratio = 1) using a Partial-ROC criterion.

+ indicates the overlapped area related with at least one of the ENMs generated by species within the *Pristimantis acuminatus* complex.

### Ecological niche equivalence

Warren's Identity test revealed that most pairs of ENMs are not equivalent (i.e., identical), but are nonetheless highly similar (Table 6). Schoener’s *D* is large for comparisons among the ENMs for clades A, B, and D (0.69–0.75), but small for comparisons of clade C to all other clades (0.56–0.67) (Table 6). All observed niche overlap values were significantly smaller than those of the null models in the niche Identity Tests (*P* < 0.05), except for clades A vs. B and A vs. D (*P* = 0.09). High niche similarity index values (*D* ≥ 0.69) among ENMs for clades A, B and D are mainly influenced by annual precipitation and extreme environmental factors (e.g. Precipitation of the Driest Month Wettest Quarter, and Warmest Quarter). Only *P*. *acuminatus* has moderate overlap values for ENMs (*D* = 0.56–0.67), which are mainly related to Monthly Precipitation Seasonality (S3 Fig).

**Table 6:** Niche overlap (Schoener's D index) and Identity Test for clades within *Pristimantis acuminatus* complex.

Statistics	A–B	A–C	A–D	B–C	B–D	C–D
Schoener's D index	0.75	0.56	0.72	0.59	0.69	0.67
Null model						
Mean	0.83	0.80	0.79	0.72	0.82	0.79
Standard deviation	0.05	0.05	0.05	0.05	0.04	0.05
Minimum	0.66	0.70	0.63	0.59	0.68	0.65
Maximum	0.93	0.91	0.88	0.80	0.90	0.91
*P-value*	0.09	<0.01	0.09	0.01	0.01	0.01

D-values are compared to the null distributions for each pair of species (clades A–D); P-values are provided for each comparison.

### Taxonomic considerations

**Fig 5:**
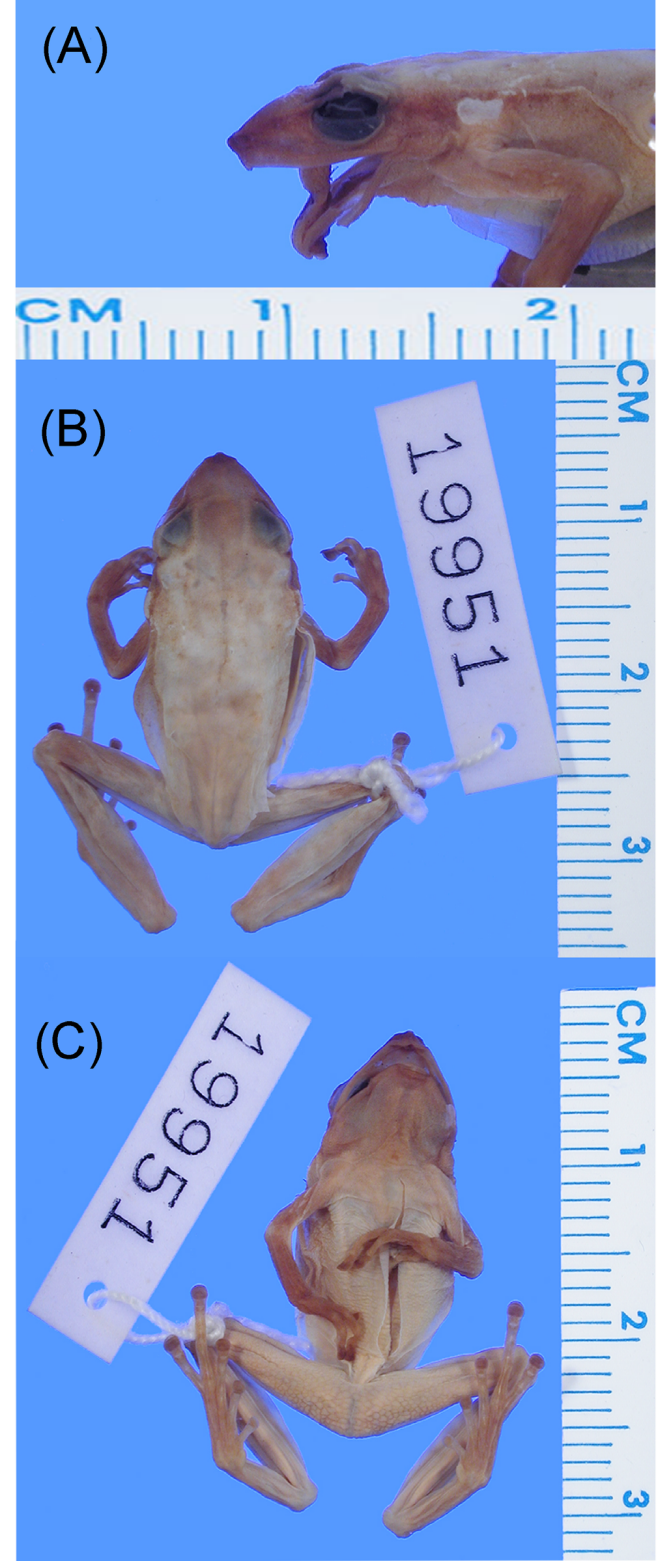
Holotype of *Pristimantis acuminatus* (MCZ 19951, A: lateral view of head, B: dorsum, C: venter). This specimen was collected in Canelos, Pastaza province, Ecuador. Note the shagreened dorsum and absence of tympanum. Photographs by the President and Fellows of Harvard College, Museum of Comparative Zoology at Harvard University.

The available name for the populations sampled in our phylogeny is *Pristimantis acuminatus* Shreve [42]. The holotype (MCZ A19951) is an adult male (SVL = 22.13 mm) in a relative good status of conservation (Fig 5), collected from the southern lowlands in the Amazon basin of Ecuador. Regarding comparisons with the holotype of *Pristimantis acuminatus*, integrative information from combined genetic, morphological, distributional and ecological evidence, we assign this name to clade C in our phylogeny (Figs 1 and 2, Table 2). The holotype share with specimens from clade C in our phylogeny, the following specific diagnostic characters: 1) the tympanic annulus is not visible on skin (= tympanum absent, sensu [30]), 2) tarsus lacking folds or tubercles, 3) skin on dorsum shagreened, 4) lacking supernumerary plantar tubercles, and 5) bearing short vocal slits, vocal sac small and nuptial excrescences cream.

**Fig 6:**
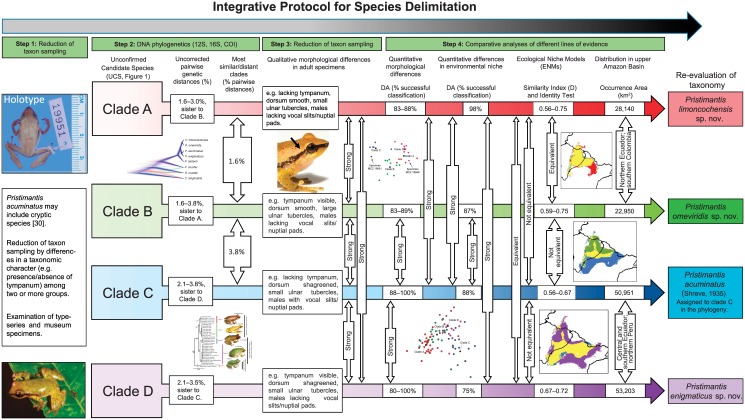
Schematic working protocol for an integrative systematics. Increasing black color intensity represents increasing certainty about species status in the *Pristimantis acuminatus* complex [48]. Colors in species represent clades shown in the phylogeny and geographic ranges in the Fig 1.

The main diagnostic characters used herein to distinguish *P*. *acuminatus* from other species in the complex are: 1) the tympanic annulus is not visible on skin, 2) tarsus lacking tubercles or folds, 3) genetic p-distances ranging from 1.8–4.7% (3.3±2.1) for 12S, 2.1–3.8% (2.96±0.9) for 16S gene, and 4.7–7.9% (6.7±1.8) for COI. Accordingly, the northern Amazonian populations of the *Pristimantis acuminatus* complex (clades A and B), and southern populations (clade D) in Ecuador and Peru require formal description as different species. Based on the current systematic work, we provide a schematic working protocol and diagnostic characters to delimit these species (Fig 6 and Table 7).

**Table 7:** Comparisons of *Pristimantis enigmaticus* sp. nov., *P*. *limoncochensis* sp. nov., and *P*. *omeviridis* sp. nov. with other species from the Amazonian lowlands having a dorsal green coloration.

Diagnostic character	*P*. *acuminatus*	*P*. *enigmaticus sp*. *nov*.	*P*. *limoncochensis sp*. *nov*.	*P*. *olivaceus*	*P*. *omeviridis sp*. *nov*.	*P*. *padiali*	*P*. *pseudoacuminatus*	*P*. *tantanti*
Dorsal skin texture	Shagreened	Shagreened	Smooth	Shagreened with scattered tubercles	Smooth	Shagreened	Shagreened, with scattered warts and/or tubercles	Shagreened
Ventral skin texture	Areolate	Areolate	Areolate	Areolate	Coarsely areolate	Areolate	Coarsely areolate	Areolate
Tympanic annulus beneath skin	—	+	—	+	+	—	+	—
Vocal slits	+	—	—	+	—	—	+	—
Nuptial pads	+	—	—	+	—	—	—	—
Discoidal fold	+	—	+	+	+	+	+	—
Ulnar tubercles	Small	Small	Small	Small	Large	Forming a row	Small	Tubercles coalescing into a fold
Tarsal fold or tubercles	—	Small tarsal fold	Small tubercles	Small tubercles	Large tubercles	Large tarsal fold	Small tubercle	Tubercles coalescing into a fold
Relative size inner/outer metatarsal tubercles	5x	4x	3x	3x	2x	4x	4x	2x
Supernumerary plantar tubercles	—	+	+	+	+	+	+	+
Dorsal coloration in life	Bright greenish yellow with or without scattered black, orange or brown blotches	Bright greenish yellow, with or without scattered black and orange blotches	Bright greenish yellow with or without scattered black, orange or brown blotches	Olive green to yellowish green, usually with few dark brown to black spots	Bright greenish yellow with or without scattered black, orange or brown blotches	Bright green to yellowish green with white spots	Green marbled with brown or chevrons	Green with white spots
Vertical bars below eye	—	—	—	—	—	—	+	—
Belly coloration in life	Yellowish cream	Pale cream to white	Bright yellow to cream	cream yellow	Pale cream to white	Yellow	Green with small white spots	Greenish yellow
Throat coloration in life	Yellowish tan	Pale cream to white	Greenish tan	Yellow	Pale cream to white	White to yellow	White with brown suffusion	Greenish yellow
Plantar and palmar surfaces coloration in adults	Yellowish tan	Tan	Greenish tan	Greenish yellow	Tan	Dark brown	Greenish brown	Yellow to yellowish brown
Iris coloration	Coppery red	Coppery red, finely reticulated with black	Coppery red	Bronze with fine black reticulation	Bronze, finely reticulated with black	Light reddish brown	Bronze, finely reticulated with black	pale beige, with fine brown reticulation

Characters were taken from original species description and from specimens examined. Presence of a character is indicated by “+”, its absence by “−”.

### Species Accounts

Class AMPHIBIA Linnaeus, 1758

Order ANURA Fischer von Waldheim, 1813

Family CRAUGASTORIDAE Hedges, Duellman, and Heinicke, 2008

Genus *Pristimantis* Jiménez de la Espada, 1870

#### *Pristimantis acuminatus* (Shreve 1935)

*Eleutherodactylus acuminatus*.—Shreve, 1935, Occasional Papers of the Boston Society of Natural History, 8: 217.

*Pristimantis acuminatus*.—Heinicke, Duellman, and Hedges, 2007, Proceedings of the National Academy of Sciences. USA, Supplementary Information, 104: Table 3.

**Holotype:** MCZ 19951, by original designation. Collected April 1932 by O.C. Felton from Canelos, Pastaza Province, Ecuador.

**Characteristics:** *Pristimantis acuminatus* is characterized by: (1) skin of dorsum shagreened, with scattered small tubercles in males; dorsolateral folds absent; skin of belly areolate; discoidal fold prominent; (2) tympanic annulus and membrane not visible, covered by muscle beneath skin; (3) snout long, acuminate (females) to subacuminate (males) in dorsal view, truncated and posteriorly inclined in profile, bearing a rostral papilla in some; lips flared, canthus rostralis angular in dorsal and lateral view, loreal region concave; (4) upper eyelid about 60% of inter-orbital distance, lacking tubercles; (5) dentigerous processes of vomer small, triangular, bearing 3–4 teeth; (6) males with short vocal slits, extending from mid-lateral base of tongue to the angle of the jaw; vocal sac small, nuptial excrescences cream; (7) fingers large and slender, first shorter than second; discs on outer fingers expanded, bluntly truncated, about 1.5x the width of digit proximal to pad; supernumerary tubercles prominent, rounded; (8) fingers bearing lateral fringes; (9) forearm bearing 1–4 ulnar tubercles, small; (10) heel lacking tubercles; inner and outer border of tarsus smooth; tarsal folds absent; (11) two metatarsal tubercles; inner elliptical, about 5x the outer tubercle; supernumerary plantar tubercles absent; (12) toes with lateral fringes; webbing absent; discs equal in size or slightly smaller than those on fingers; Toe V longer than Toe III; (13) in life, dorsum bright greenish yellow with scattered black, orange or brown blotches; groin and anterior surfaces of thighs uniformly yellowish; ventral surfaces of belly yellowish cream; throat, foot and hand yellowish tan; black canthal stripe extending from eye to mid-flank; iris coppery red. In preservative, all yellowish areas fade to cream; anterior and posterior surfaces of thighs uniformly tan; venter cream; canthal area and snout black; (14) SVL in adult males 22.8±1.12 mm standard deviation (20.91–24.01 mm); females with 30.5±2.96 mm (27.1–33.45 mm).

**Diagnosis:** Among greenish Amazonian *Pristimantis*, *P*. *acuminatus* is most similar to *P*. *limoncochensis* sp. nov., *P*. *padiali*, and *P*. *tantanti* by lacking tympanum and an externally visible tympanic annulus (it is concealed by muscle). Ulnar and tarsal tubercles in *P*. *acuminatus* are small or absent, whereas they are prominent in a row in *P*. *padiali* or coalescing forming a fold in *P*. *tantanti*. Furthermore, *P*. *padiali* have a dark brown plantar and palmar coloration (tan in *P*. *acuminatus*), whereas the relative size among inner/outer metatarsal tubercles is 2x smaller in *P*. *tantanti* compared with the 5x in *P*. *acuminatus*. *P*. *acuminatus* and *P*. *limoncochensis* sp. nov. having small ulnar tubercles (not forming a row or fold), but tarsal and supernumerary plantar tubercles are absent in the former species. Furthermore, dorsal skin is shagreened in *P*. *acuminatus*, but smooth in *P*. *limoncochensis* sp. nov. Males in *Pristimantis acuminatus* have vocal slits and nuptial pads, which are absent in males of *P*. *limoncochensis* sp. nov., *P*. *padiali* and *P*. *tantanti* (Table 7). Comparisons of diagnostic characters between *Pristimantis acuminatus* and the new species are detailed in Table 2.

**Variation:** Measurements and proportions are provided in Table 3. Sexual dimorphism is evident in this species, males are smaller (22.8±1.12 mm; 20.91–24.01 mm) than females (30.5±2.96 mm; 27.1–33.45 mm). Furthermore, adult females present a well-defined acuminate shape in snout, compared with the subacuminate snout in males. In life, most specimens present a well-defined canthal stripe, which is less visible in preserved material, with a dark brown to black snout (e.g. QCAZ 53263, 53845). The holotype has a small tubercle (rostral papilla) on the tip of snout which is variable in prominence among specimens.

**Coloration in life:** Body coloration is darkest in specimens observed by day, and brightest in specimens observed at night. At night, dorsum and flanks are bright greenish yellow; black dots and orange blotches are present on dorsum; the legs of males are densely flecked. Interobital bar, subocular stripes, scapular and sacral marks absent; sides of head colored as dorsum, black canthal stripe continuing until the mid-flank; groin and anterior surfaces of thighs yellowish white; posterior surfaces of thighs uniform yellowish cream; ventral surfaces of belly yellowish cream; throat, foot and hand yellowish tan. Dorsal surfaces of pads on Fingers II and III distinctly yellow, dark brown on Fingers III and IV; iris coppery red. By day, all bright surfaces turn into dark greenish yellow.

**Coloration in preservative:** Dorsum cream, stippled with minute brown flecks. Sides of head dark brown to black, canthal stripe barely visible. Forearms and hind limbs with barely defined dark brown marks. Venter, throat, chest, ventral surfaces of limbs, and palms, cream, densely stippled with minute brown flecks (visible under magnification); posterior surfaces of tarsus and plantar surfaces uniform cream.

**Natural history and distribution:** *Pristimantis acuminatus* is known from 13 localities along Amazonian evergreen lowland forest of southern Ecuador in Pastaza and Morona-Santiago Provinces, and five localities from northern Loreto Department in Peru, up to 1123 m elevation. Specimens examined, but not included in morphometric or phylogenetic analyses, from Leticia [7–11 km road to Tarapacá (ICN 11187)] and from Parque Nacional Amacayacu (IAVH 4628), Amazonas Department are tentatively assigned to this species (Fig 1). The area of known occurrence is calculated to be about 50,951 km2 in lowland and piedmont evergreen forest in eastern Ecuador and Peru, but a wider distribution is likely, up to 201,174 km2 in the upper Amazon Basin (Fig 4). According to field notes and museum databases, specimens of *P*. *acuminatus* were found active at night on leaves of low vegetation, up to 3 m above ground. It is suspected that is an inhabitant on forest canopy. Calling and reproductive behavior are unknown.

**Remarks:** According with our phylogenetic analyses, *P*. *acuminatus* is sister to *P*. *enigmaticus* from populations along the southern Amazonia of Ecuador and northern Peru.

#### *Pristimantis enigmaticus* sp. nov. Ortega-Andrade, Rojas-Soto, Valencia, Espinosa de los Monteros, Morrone, Ron, and Cannatella

urn:lsid:zoobank.org:act:A23E7AA5-15EE-4448-B13E-6B58532A65CA

(Figs 7–10)

**Fig 7:**
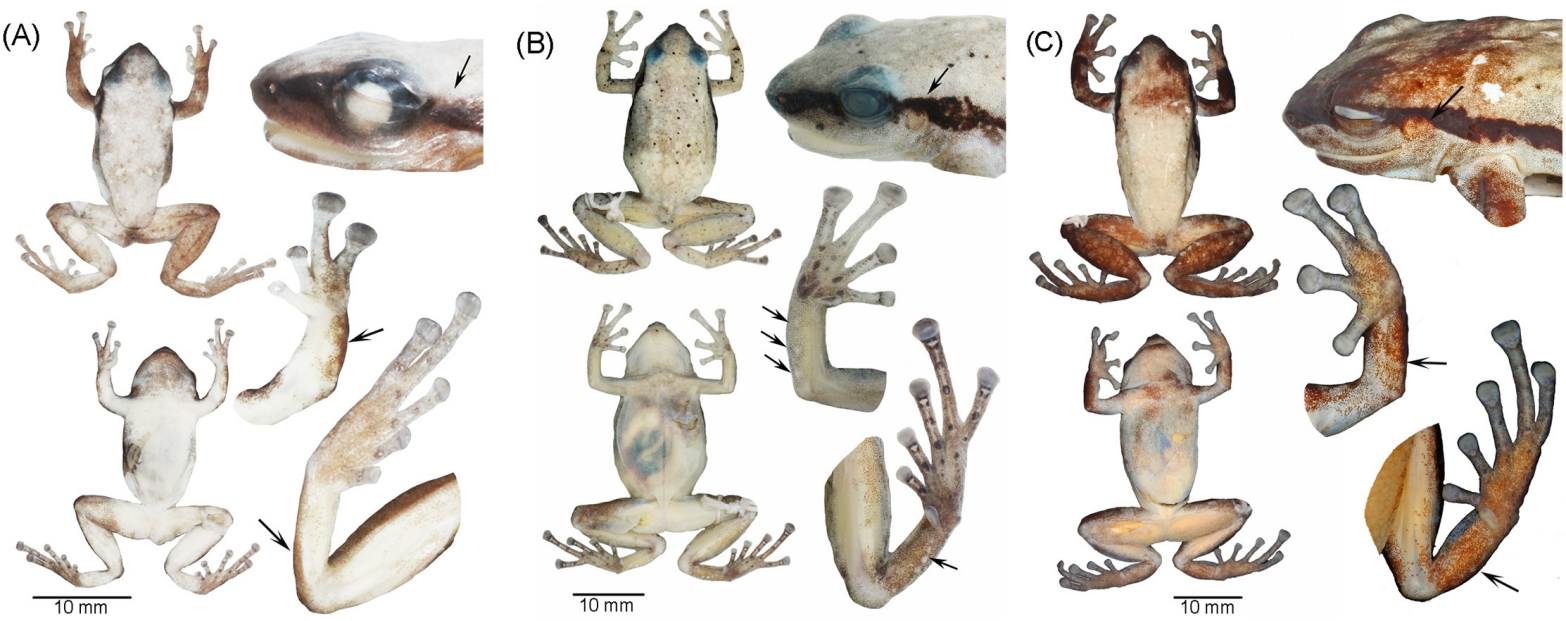
Holotypes of the new species. Views of the body (dorsum and venter), head, hand, and foot of the holotypes of **(A)** *Pristimantis limoncochensis*, QCAZ 37277, **(B)** *Pristimantis omeviridis*, QCAZ 55392, and **(C)** *Pristimantis enigmaticus*, QCAZ 40935. Arrows indicate the absence (*Pristimantis limoncochensis*) or presence of tympanum (*Pristimantis omeviridis* and *Pristimantis enigmaticus*), and differences in ulnar and tarsal tubercles on arms and legs. Tags and background color have been digitally removed. Only the dorsum and venter are shown at scale. Photographs by H. M. Ortega-Andrade.

**Fig 8:**
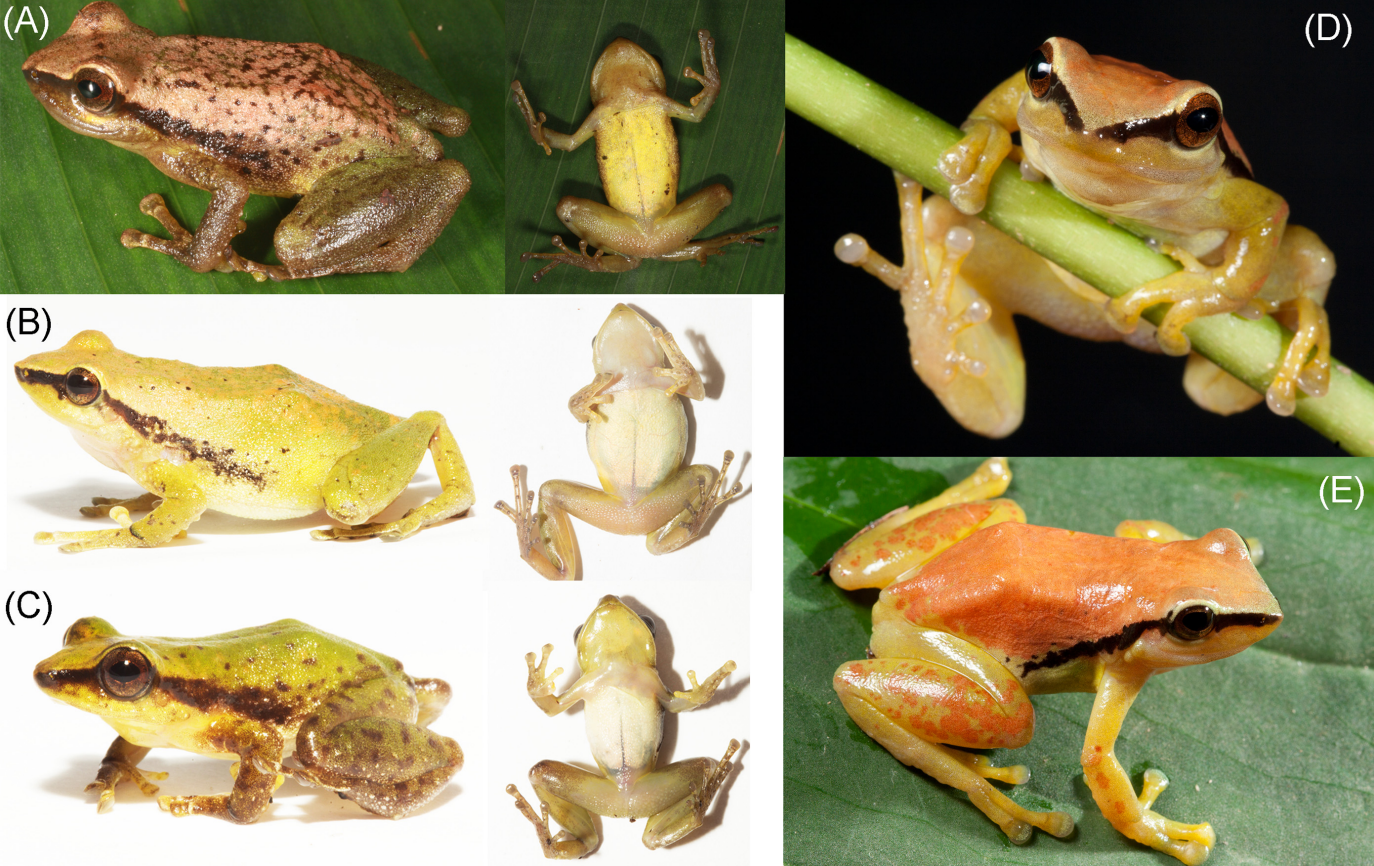
Live specimens of the new species (A: *Pristimantis limoncochensis* sp. nov., B-C: *Pristimantis omeviridis* sp. nov., D-E: *Pristimantis enigmaticus* sp. nov.). **(A)** Paratype female, QCAZ 52987; **(B)** holotype female, QCAZ 55392; **(C)** paratype male, QCAZ 55391; **(D-E)** holotype female, QCAZ 40935. Photographs by H. M. Ortega-Andrade **(A)** and S. Ron **(B-E).**

**Fig 9:**
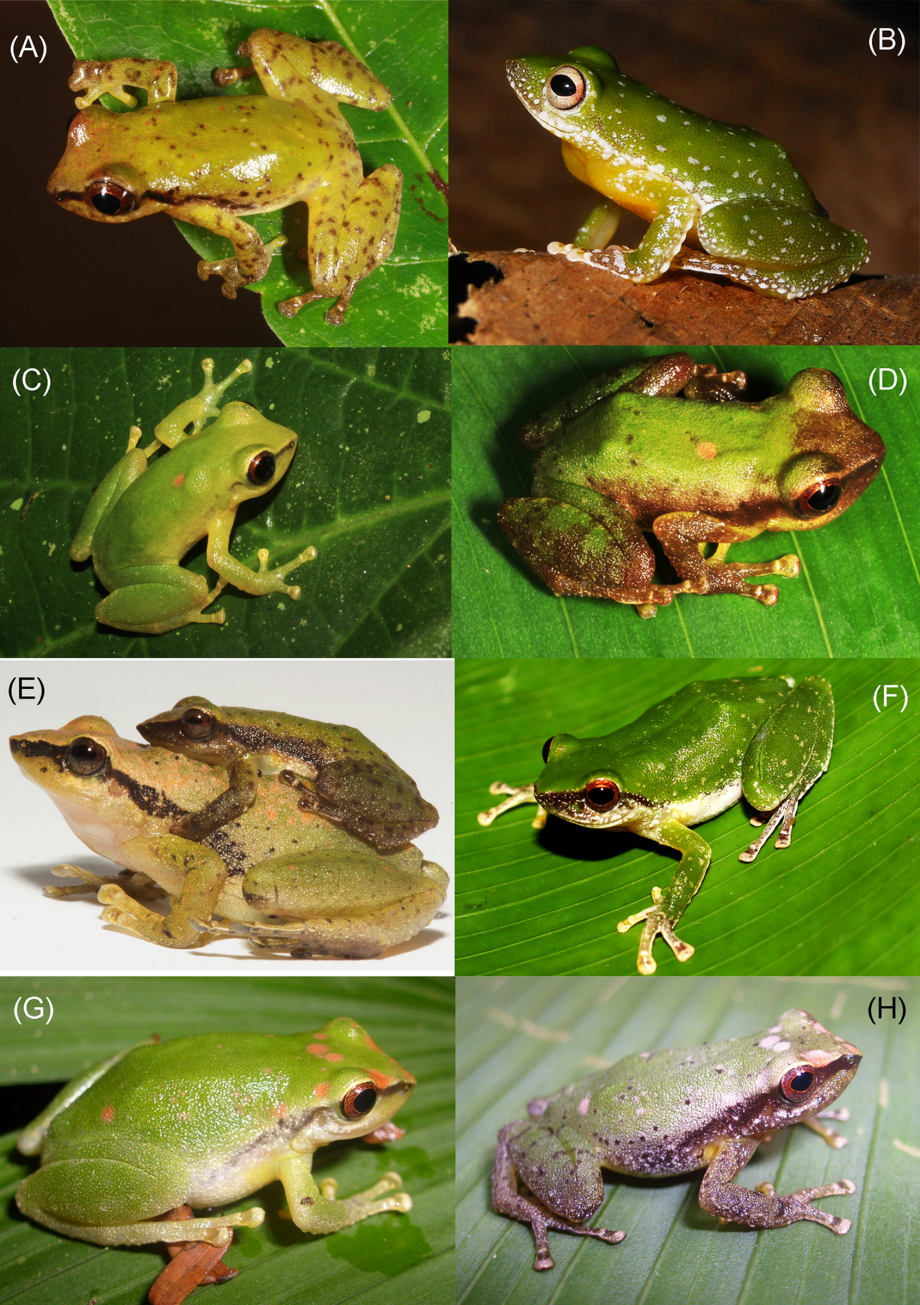
Living specimens of the *Pristimantis acuminatus* complex and their relatives in the Amazon Basin. **(A)** *Pristimantis acuminatus*, QCAZ 53263, **(B)** *Pristimantis tantanti*, CORBIDI 12987, **(C-D)** night and daylight color variation in *Pristimantis limoncochensis* sp. nov., QCAZ 52987, **(E)** amplectant pair of *Pristimantis omeviridis* sp. nov., holotype female QCAZ 55392 and paratype male QCAZ 55391, **(F)** *Pristimantis padiali*, specimen not collected, **(G-H)** night and daylight color variation in *Pristimantis enigmaticus*, specimen not collected. Photographs of **(B)** by V. Durán, **(E)** by Santiago Ron, **(F)** by Omar Rojas; all other photographs by H. M. Ortega-Andrade.

**Fig 10:**
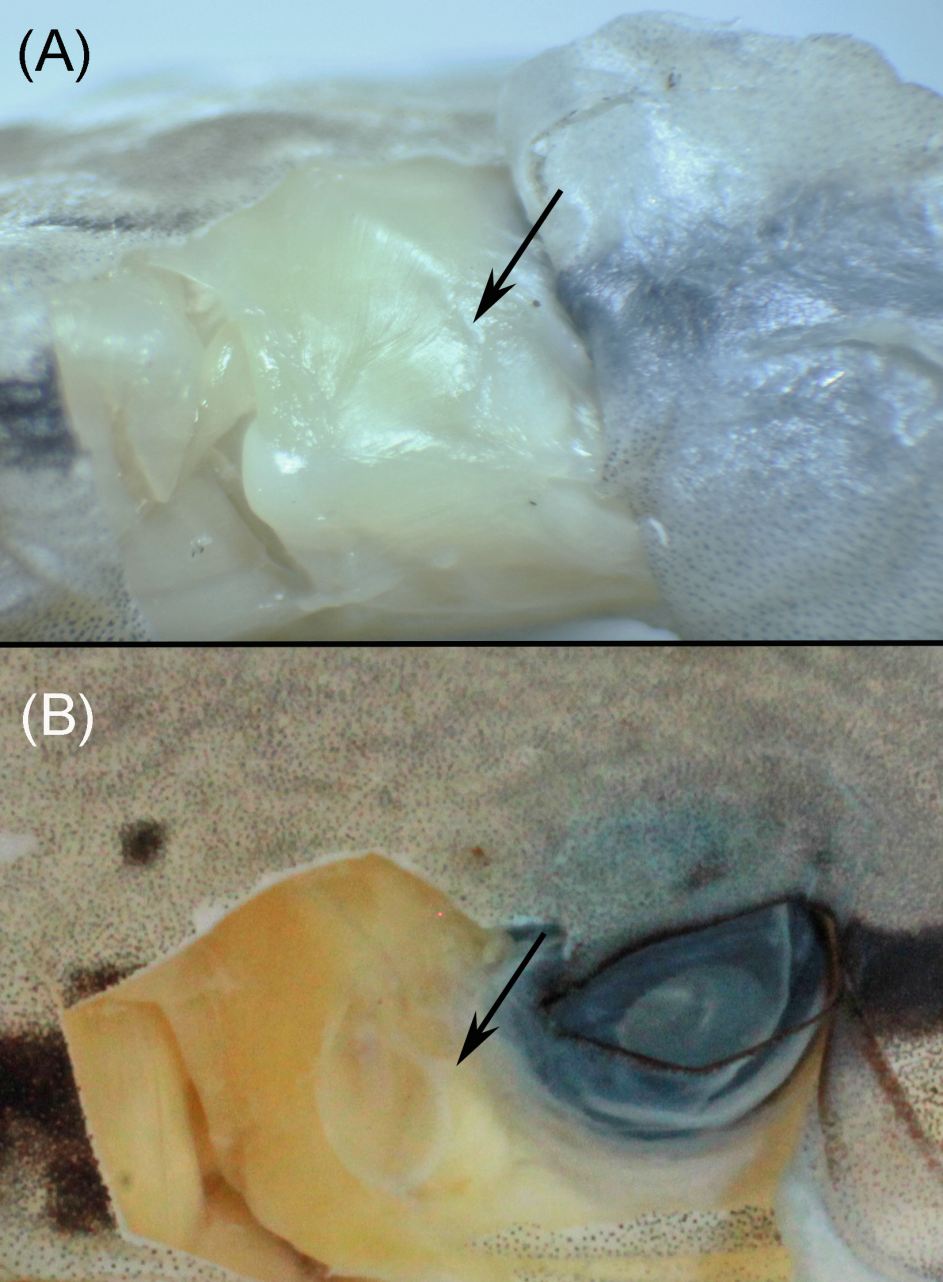
Variation in tympanum condition. **(A)** Tympanum covered by muscle, overlying skin not differentiated (*Pristimantis limoncochensis* sp. nov., QCAZ 52987); **(B)** tympanic annulus and membrane distinct, not covered by muscle (*Pristimantis enigmaticus* sp. nov., QCAZ 31184), overlying skin is differentiated (not shown in photo). Photographs by H. M. Ortega-Andrade.

*Eleutherodactylus acuminatus—*Shreve, 1935, Occasional Papers of the Boston Society of Natural History, 8: 217.

*Pristimantis acuminatus—*Heinicke, Duellman, and Hedges, 2007, Proceedings of the National Academy of Sciences. USA, Supplementary Information, 104: Table 3.

*Pristimantis* cf. *acuminatus*—Duellman and Lehr, [30]. Nature und Tier Verlag, Münster, Germany: 370.

*Pristimantis acuminatus*—Ortega-Andrade, [88]. Dissertation Thesis. Instituto de Ecología, A.C.: 103: Appendix I.

**Holotype:** QCAZ 40935, an adult female collected on 11 March 2009 at 6 km on road San Ramón−El Triunfo, Cooperativa La Mariscal Sucre, 500 m in the trail of Pukayacu river, S°1.370, W°77.860, 950 m elevation, by Santiago R. Ron, Italo G. Tapia, Luis A. Coloma, Amaranta Carvajal-Campos and Andrés Tapia, Orellana Province, Ecuador.

**Paratypes:** ECUADOR: Morona Santiago province: QCAZ 32522 collected on 19 August 2006 at Bobonaza, Tuculí road, S°1.4945, W°77.8697, 653 m; QCAZ 48669 collected on 13 June 2010 at Comunidad San Luis, S°3.3421, W°78.4677, 775 m. Orellana province: QCAZ 54275 collected on 19 September 2012 at Boamano, S°1.2638, W°76.3623, 229 m, by Morley Read; QCAZ 40496 collected on 1 December 2008 at Parque Nacional Yasuní, Plataforma Daimi A, S°0.9929, W°76.2037, 240 m. Pastaza province: QCAZ 38559 collected on 28 January 2008 at Alrededor de Villano, AGIP, oil camp Villano B Unidad 1, S°1.453, W°77.4437, 367m; QCAZ 38771 collected on 5 December 2008 near Villano, AGIP oil camp, Villano B-II Unidad 3, S°1.4557, W°77.4447, 367 m, by Yadira Mera, Diego Paucar, Fernando Ayala-Varela; QCAZ 39030 collected on 30 July 2008 at Alrededores de Villano, Comunidad Kurintza, S°1.5042, W°77.5143, 405m; QCAZ 39438, 39445 collected on 12 October 2008 at Bataburo Lodge, al sur de la carretera desde Cononaco, S°1.2083, W°76.7167, 241m; QCAZ 54951 collected on 30 November 2012 at Campo Villano (AGIP). Villano A, S°1.471, W°77.4517, 420 m, by Andrea Narváez; QCAZ 52953 collected on 26 March 2012 at Comunidad Santa Rosa, S°2.0809, W°76.9347, 297 m, by Freddy F. Velásquez-Alomoto, David Toquetón, Libio Santi and Jaime Manya; QCAZ 40936 collected on 22 March 2008 at km 6 on road from San Ramón-El Triunfo, closest town to Colonia Mariscal Sucre, Centro Ecológico Sancha Arajuno, S°1.3533, W°77.8645, 956; QCAZ 40918 collected on 11 April 2009 at km 6 on road from San Ramón-El Triunfo, Cooperativa La Mariscal Sucre, 500 m before Río Pucayaku, S°1.37, W°77.86, 950 m, by Santiago R. Ron, Italo G. Tapia, Luis A. Coloma, Amaranta Carvajal-Campos, Andrés Tapia; QCAZ 31184 collected on 1 June 2006 at Parroquia Teniente Hugo Ortíz km 6 on road from San Ramón- El Triunfo Colonia Mariscal Sucre (Hacienda Alejandra, zoocriadero Fátima), S°1.3541, W°77.8616, 939 m, by Omar Torres-Carvajal and Stephanie Swenson; QCAZ 33222 collected on 30 March 2007 at Pomona, Estación Hola Vida, S°1.625, W°77.9072, 821 m, by Italo G. Tapia, Diego Almeida-Reinoso, Monica Paez; QCAZ 48656 collected on 24 June 2010 at San Juan de Piatua, S°1.1997, W°77.9502, 832m; MCZ A19949 collected on 1 January 1933 at Sarayacu, S°1.6779, W°77.4815, 474 m, by O.C. Felton and T. Barbour; FHGO 341, 494 collected at Villano, S°1.5, W°77.47, 393 m. PERU: Loreto department: CORBIDI 4720 collected on 1 March 2008 at Andoas, S°2.3511, W°75.8162, 173 m, by Vilma Duran; CORBIDI 1128 collected on 1 September 2008 at San Jacinto, S°2.3308, W°75.8637, 177 m, by Amanda Delgado; CORBIDI 6449 collected at Shiviyacu, S°2.4819, W°76.0857, 218 m, by Amanda Delgado.

**Characteristics:** *Pristimantis enigmaticus* is characterized by: (1) skin of dorsum shagreened; dorsolateral folds absent; skin of belly areolate; discoidal fold absent; (2) tympanic annulus and membrane visible; (3) snout long, acuminate in dorsal view, bearing a pointed papilla in adult females; truncated and posteriorly inclined in profile; lips not flared, canthus rostralis angular in dorsal and lateral view, loreal region concave; (4) upper eyelid about 60% of inter-orbital distance, lacking tubercles; (5) dentigerous processes of vomer large, transverse, bearing 6–8 teeth; (6) males lacking vocal slits, vocal sac, and nuptial excrescences; (7) fingers large and slender, first shorter than second; discs on outer fingers expanded, bluntly rounded, about 1.5x the width of digit proximal to pad; supernumerary tubercles barely visible, small, rounded; (8) fingers bearing lateral fringes; (9) forearm bearing 2–3 ulnar tubercles, small; (10) heel lacking tubercles; outer border of tarsus smooth; inner border of tarsus bearing a small tarsal fold; (11) two metatarsal tubercles; inner elliptical, about 4x the outer tubercle; lacking supernumerary plantar tubercles; (12) toes with lateral fringes; webbing absent; discs equal in size than those on fingers; Toe V longer than Toe III; (13) in life, dorsum bright greenish yellow or pale orange to pink, with or without scattered blackish blotches; groin and anterior surfaces of thighs uniformly pale yellow; belly and throat pale cream to white; ventral surfaces of hands and feet tan; black canthal stripe continuing posterior to the eyes, reaching mid-flank; iris coppery red, finely reticulated with black. In preservative, all yellow or green areas fade into cream or tan; anterior and posterior surfaces of thighs uniformly tan; venter cream, throat finely stippled with minute brown flecks; black canthal stripe; (14) SVL in adult males 20.7±2.32 mm (18.51–24.79 mm); females 30.8±2.92 mm (26.38–36.37 mm).

**Diagnosis:** Among greenish Amazonian *Pristimantis*, *P*. *enigmaticus* is most similar to *P*. *omeviridis* sp. nov., *P*. *pseudoacuminatus* and *P*. *olivaceus* in having a differentiated tympanic annulus and tympanum. *Pristimantis enigmaticus* is like *P*. *omeviridis* (characters in parentheses) in coloration and snout shape, but the former species differs by having a shagreened dorsum (smooth), lacking discoidal fold (present), bearing small ulnar tubercles (large) and tarsal fold (tubercles). Males of *P*. *pseudoacuminatus* and *P*. *olivaceus* have vocal slits and nuptial pads, which are absent in males of *P*. *enigmaticus*. Furthermore, the smaller *P*. *pseudoacuminatus* has a distinctive discoidal fold, brown vertical bars below eye, and tubercles on dorsum and heel; these are absent in *P*. *enigmaticus*. *Pristimantis olivaceus* is distributed in the southern Amazon Basin, and differs from *P*. *enigmaticus* in having an olive body coloration (greenish yellow in *P*. *enigmaticus*), having small tarsal tubercles (tarsal fold) and by a distinctive discoidal fold (absent).

**Description of the holotype:** Body slender; head wider than body; slightly longer than wide, about 30% of SVL; snout long, acuminate in dorsal view, truncated and posteriorly inclined in profile; distance from nostril to corner of eye equal in length to diameter of eye; *canthus rostralis* straight in dorsal view, angular in cross section, sloping gradually to lips; lips not flared; internarial area not depressed, nostrils not protuberant, directed anterolaterally, situated about three-quarters distance from eyes to tip of the snout; interorbital area flat, IOD 40% of head width; eye large, protuberant, its diameter about 3x depth of lip below eye, and about 30% of head length; upper eyelid about 65% of inter-orbital distance, lacking tubercles; no interocular fold; cranial crests absent. Tympanic membrane and annulus prominent, round in shape, with supratympanic fold partially obscuring upper and posterodorsal edges; horizontal diameter of tympanum 30% of eye diameter, separated from eye by a distance of one-third tympanum length; postrictal tubercles constricted and fused to form a barely visible short ridge extending ventrolaterally from tympanum; choana small, rounded, not concealed by palatal shelf of maxillary arc; dentigerous processes transverse, angled postero-medially and closely separated; bearing eight small teeth; tongue elliptical, posterior border notched, not adherent to floor of mouth for about 40% of its length.

Skin on dorsum shagreened; no occipital ridges or dorsolateral folds; skin on flanks shagreened to areolate; ventral surfaces of belly coarsely areolate; skin on ventral surfaces of chest, throat, and thighs smooth; discoidal folds barely visible; no thoracic fold. Forearm slender; fingers large and slender, all with oval (broader than long) pads, Fingers III-IV with large pads, all fingers with large discs; pad on Finger III about 1.5x wider than narrowest portion of penultimate phalanx; disc on Finger I distinctly smaller than those on other fingers; relative length of fingers I<II<IV<III; subarticular tubercles large, subconical; supernumerary tubercles prominent, subconical; palmar tubercle bifid, 2x size of oval thenar tubercle; anterbrachial tubercle small; three conical ulnar tubercles are present along inner edge of forearm; outer edge of forearm shagreened, tubercles absent;

Hind limbs slender; tibia length about 50% of SVL; knee and heel lacking tubercles; foot length about 67% of SVL; outer border of tarsus smooth; inner border of tarsus bearing four tubercles, small; inner metatarsal tubercle oval, 3x size of rounded outer tubercle; supernumerary tubercles rounded, small; subarticular tubercles subconical, rounded; toes with non-crenulate lateral fringes; webbing absent between toes; pads of Toes III−V large, in all other pads and discs of toes like those of fingers; relative lengths I<II<III<V<IV; Toe III extending to one half the distance between penultimate and ultimate subarticular tubercles on Toe IV; Toe V extending to distal edge of ultimate subarticular tubercle on Toe IV. Vent opening puckered, shagreened, not extended, lacking tubercles on its border, located at upper level of thighs.

**Measurements (in mm) of the holotype:** Specimen QCAZ 40935 is an adult female with the following measurements: SVL = 34.4; HL = 11.6; HW = 12.2; ED = 4.0; EN = 3.9; IOD = 4.5; EW = 2.2; TD = 1.5; FL = 15.9; TL = 16.4; FtL = 22.5; HdL = 9.8; F3 = 2.1; T4 = 1.9. Proportions: HL/SVL = 0.3; HW/HL = 1.1; FL/SVL = 0.5; TL/SVL = 0.5; FtL/SVL = 0.7; EN/HL = 0.3; ED/HL = 0.3; IOD/HW = 0.4; TD/ED = 0.4.

**Variation:** Measurements and proportions of specimens examined are in Table 3. Sexual dimorphism is evident in this species, males being smaller (20.7±2.32 mm; 18.51–24.79) than females (30.8±2.92 mm; 26.38–36.37). Furthermore, adult females present a well-defined acuminate snout, bearing a small papilla. Males vary in snout shape, from sub-acuminate to acuminate. Ulnar tubercles are visible in adult males and females, but absent in juveniles. In life, most specimens show chromatic variation, being bright green by night and dark greenish brown by day.

**Coloration in life** (Figs 8 and 9): At night, dorsum bright green with or without black dots and orange blotches; flanks greenish white. Interobital bar, subocular stripes, scapular and sacral marks absent; sides of head colored as flanks, black canthal stripe continuing until reach the mid-flank; groin and anterior surfaces of thighs yellowish white; posterior surfaces of thighs uniform yellowish cream; ventral surfaces of belly yellowish cream; throat, foot and hand yellowish tan. Dorsal surfaces of pads on Fingers II and III distinctively cream white; iris coppery red. By day, all bright surfaces turn into dark greenish, orange blotches to pink; flanks, dorsal surfaces of limbs turning to dark brown with white flecks.

**Coloration in preservative:** Dorsum cream or tan with or without brown flecks. Sides of head similar to dorsum, with a well-defined canthal stripe. Forearms and hind limbs with barely defined dark brown marks. Venter, throat, chest, ventral surfaces of limbs, and palms, cream densely stippled with minute brown flecks (visible under magnification); posterior surfaces of tarsus and plantar surfaces uniform cream.

**Etymology:** The specific name is derived from the Latin adjective “aenigmaticus” (“puzzling, obscure, enigmatic”) and refers to the fact that this species was hidden for decades in a complex of cryptic species. The epithet is an adjective.

**Natural history and distribution:** *Pristimantis enigmaticus* is known from 17 localities along Amazonian evergreen lowlands and piedmont of southern Ecuador in Morona-Santiago, Orellana and Pastaza Provinces, and five localities from northern Loreto Department in Peru, up to 960 m elevation. The area of occurrence is calculated to be about 53,202 km^2^ in lowland and piedmont evergreen forest in eastern Ecuador and Peru, but a wider distribution is likely, up to 370,019 km^2^ in the upper Amazon Basin (Fig 4). According to field notes and database records, specimens of *Pristimantis enigmaticus* were found active at night on leaves of low vegetation, up to 3 m above ground, but it is suspected that it is an inhabitant of forest canopy. Calls and reproductive behavior are unknown.

**Remarks:** Lynch and Duellman [89] proposed and discussed varying conditions of tympanum, which were widely followed in works of systematics and taxonomy of direct-developing frogs [29,30,90]. Following several important papers in the upper Amazon Basin of Ecuador and Peru [20,30,45], the diagnostic character of “Tympanum concealed beneath skin” was frequently applied to separate this species from congeners [43,44]. However, as noted in the original species description, the holotype of *P*. *acuminatus* (MCZ 19951) lacks a defined tympanic annulus, which is present on two paratypes (MCZ A19949-50); we re-identified them as *Pristimantis enigmaticus* sp. nov. According to the phylogenetic analyses, *P*. *enigmaticus* is sister to *P*. *acuminatus*. Specimens CORBIDI 2537 (Sierra del Divisor, S°6.21361, W°73.2391, 500 m, Loreto, Peru) and MHNC 11178 (Reserva Comunal Machiguenga, S°12.1789, W°73.0814, Cusco, Peru) were not included in phylogenetic analyses, but match most of the morphological traits described for *P*. *enigmaticus*, except for the rounded shape of snout. Both specimens are tentatively considered representatives of different species in southern and central Peru, maybe related to *P*. *olivaceous* [91].

#### *Pristimantis limoncochensis* sp. nov. Ortega-Andrade, Rojas-Soto, Valencia, Espinosa de los Monteros, Morrone, Ron, and Cannatella

urn:lsid:zoobank.org:act:A0F026EA-05AB-4E3A-928D-AD0B35BE7112

(Figs 7–10)

*Eleutherodactylus acuminatus*.—Duellman [20]; Lynch [45].

*Pristimantis acuminatus*.—Beirne and Whitworth [92].

**Holotype:** QCAZ 37277, an adult male collected on 27 February 2007 at Bosque Protector Pañacocha, S°0.427, W°76.05, 250 m elevation, by Silvia Aldás Alarcón, Sucumbíos Province, Ecuador.

**Paratypes:** Eight adult males (FHGO 7001, QCAZ 11996, 19180, 25814, 30937, 56316, 7095, 7097), six gravid females (QCAZ 11995, 29246, 30954, 40561, 7094, 9471) and four juvenile females (FHGO 9265, QCAZ 7096, 8521, 52987), all collected from four localities along northern Amazonian lowlands of Ecuador. Napo Province: QCAZ 30937 and 30954 collected at Huino, S°0.647781, W°77.144834, 273 m on 6 February 2003; QCAZ 7094–97 from Río Huataraco, 70 km east from Hollín, S°0.747, W°77.354, 342 m, on 15 January 1995; QCAZ 9471 from San Carlos, S°0.376347, W°76.88111, 270 m. Orellana Province: FHGO 9265 from Campo Sacha, S°0.34272, W°76.86309, 275 m, on 25 July 2013 by Manuel Dueñas. Sucumbíos Province: QCAZ 40561 from Bosque Protector Pañacocha, same collection data from holotype; QCAZ 25814 from Comunidad Asociación Chonta Yacu, Lumbaqui, S°0.1115, W°77.3743, 593 m, on 26 April 2003; QCAZ 11995–96 from Hostería La Selva, S°0.49816, W°76.3738, 249 m, on 10 April 1996 by W. Chris Funk; QCAZ 8521 from Hostería La Selva, S°0.45, W°76.28, 232 m, no other collection data; QCAZ 29246 from Laguna Grande, Reserva de Producción Faunística Cuyabeno, S°0.009701, W°76.181669, 236 m, on 13 March 2005; QCAZ 52987, 56316 from Limoncocha, S°0.40688, W°76.62063, 252 m, on 17 March 2012 and 9 May 2013 by H. Mauricio Ortega-Andrade; FHGO 7001 from Pacayacu, S°0.037895, W°76.585781, 260 m, 23 December 2008 by Miguel Alcocer; QCAZ 19180 from Saladero de Dantas, Reserva de Producción Faunística Cuyabeno, S°0.002463, W°76.177386, 215 m, on 5 February 2002 by Luis A. Coloma.

**Characteristics:** *Pristimantis limoncochensis* is characterized by: (1) skin of dorsum smooth; dorsolateral folds absent; skin of belly areolate; discoidal fold barely evident; (2) tympanic annulus and membrane not visible, covered by muscle; (3) snout long, acuminate in dorsal view, truncated and posteriorly inclined in profile; lips not flared, *canthus rostralis* angular in dorsal and lateral view, loreal region concave; (4) upper eyelid about 60% of inter-orbital distance, lacking tubercles; (5) dentigerous processes of vomer small, transverse, bearing 4–5 teeth; (6) males lack vocal slits, vocal sac and nuptial excrescences; (7) fingers large and slender, first shorter than second; discs on outer fingers expanded, bluntly truncated, about 1.5x the width of digit proximal to pad; supernumerary tubercles large, rounded; (8) fingers bearing narrow lateral fringes; (9) forearm bearing 3–5 ulnar tubercles, small; (10) heel lacking tubercles; outer border of tarsus smooth; inner border of tarsus bearing a small tarsal fold; (11) two metatarsal tubercles; inner elliptical, about 3x diameter of outer tubercle; supernumerary plantar tubercles absent or barely visible; (12) toes with lateral fringes; webbing absent; discs equal in size or slightly smaller than those on fingers; Toe V longer than Toe III; (13) in life, dorsum bright greenish yellow with or without scattered black, orange, or brown blotches; groin and anterior surfaces of thighs uniformly greenish yellowish; belly bright yellow to cream; throat, foot and hand greenish tan; black canthal stripe extending from behind the eyes until the mid-flank; iris coppery red. Well-defined yellow line present along the border or upper eyelid. In preservative, all yellowish areas fading to cream, anterior and posterior surfaces of thighs uniformly tan; venter cream; canthal stripe black, snout brown; (14) SVL in adult males 20.7±1.07 mm (18.67–22.18 mm); females with 29±1.25 mm (27.73–30.79 mm).

**Diagnosis:** Among greenish Amazonian *Pristimantis*, *P*. *limoncochensis* sp. nov. is similar to *P*. *acuminatus*, *P*. *padiali*, and *P*. *tantanti* in lacking a differentiated tympanic annulus and membrane, which are covered by muscle. Ulnar and tarsal tubercles in *P*. *limoncochensis* are small, whereas they are prominent in a row in *P*. *padiali* or coalescing forming a fold in *P*. *tantanti*. Furthermore, *P*. *padiali* has a dark brown plantar and palmar coloration (greenish tan in *P*. *limoncochensis*), whereas *P*. *tantanti* lacks a distinctive discoidal fold (present in *P*. *limoncochensis*). *P*. *limoncochensis* and *P*. *acuminatus* have small ulnar tubercles (not forming a row or fold), but tarsal and supernumerary plantar tubercles are present in the former species. Furthermore, the dorsum in *P*. *limoncochensis* is smooth compared with the shagreened dorsum in *P*. *acuminatus*, whereas males of the former species lack vocal slits and nuptial pads (both present in *P*. *acuminatus*).

**Description of the holotype:** Body slender; head wider than body; slightly longer than wide, about 40% of SVL; snout long, acuminate in dorsal view, truncated and posteriorly inclined in profile; distance from nostril to corner of eye slightly shorter than diameter of eye; *canthus rostralis* straight in dorsal view, angular in cross section, sloping gradually to lips; lips not flared; internarial area not depressed, nostrils slightly protuberant, directed anterolaterally, situated about three-quarters the distance from the eyes to the tip of the snout; interorbital area flat, IOD 43% of head width; eye large, protuberant, its diameter is about 3x depth of lip below eye and about 40% of head length; upper eyelid about 60% of inter-orbital distance, lacking tubercles; no inter-ocular fold; cranial crests absent. Tympanic annulus and membrane not visible, covered by muscle beneath skin; lacking postrictal tubercles; choana small, rounded, not concealed by the palatal shelf of maxillary arc; dentigerous processes transverse, angled postero-medially and closely separated; bearing four small teeth, visible; tongue elliptical, posterior border not notched, not adherent to the floor of the mouth for about 40% of its length; vocal slits, vocal sac and nuptial excrescences absent.

Skin on dorsum smooth; no occipital ridges or dorsolateral folds; skin on flanks shagreened; ventral surfaces of belly areolate; skin on ventral surfaces of chest, throat and thighs smooth; discoidal folds barely visible; no thoracic fold. Forearm slender; fingers large and slender, all with oval (broader than long) pads, Fingers III-IV with large pads, all fingers with large discs; pad of Finger III about 1.5x wider than narrowest portion of penultimate phalanx; disc of Finger I distinctive smaller than those on other fingers; relative length of Fingers I < II< IV< III; subarticular tubercles large, subconical; supernumerary tubercles prominent, elliptical; palmar tubercle bifid, 1.5x size of oval thenar tubercle; antebrachial tubercle small; two ulnar tubercles are present on the anterior part of forearm; outer edge of forearm shagreened, tubercles absent.

Hind limbs slender; tibia length about 53% of SVL; knee and heel lacking tubercles; foot length in about 70% of SVL; outer border of tarsus smooth; inner border of tarsus bearing a small tarsal fold; inner metatarsal tubercle oval, 1.5x size of round outer; supernumerary tubercles barely visible, rounded, small; subarticular tubercles subconical, rounded; toes with non-crenulate lateral fringes; webbing absent between toes; pads of Toes III−V large, all other pads and discs of toes like those of fingers; relative lengths I<II<III<V<IV; Toe III extending to proximal edge of penultimate subarticular tubercle of Toe IV; Toe V extending to distal edge of ultimate subarticular tubercle of Toe IV. Vent opening puckered, shagreen, not protruding, lacking tubercles on its border, located at upper level of thighs; testis cream.

**Measurements (in mm) of the holotype:** Specimen QCAZ 37277 is an adult male with following measurements: SVL = 22.18; HL = 7.8; HW = 8.16; EN = 2.55; ED = 2.55; IOD = 5.53; EW = 1.6; FL = 10.38; TL = 10.58; FtL = 15.17; HdL = 5.88; F3 = 1.27; T4 = 1.22. Proportions: HL/SVL = 0.35; HW/HL = 1.05; FL/SVL = 0.47; TL/SVL = 0.48; FtL/SVL = 0.68; EN/HL = 0.33; ED/HL = 0.33; IOD/HW 0.68.

**Variation:** Measurements and proportions of specimens examined are detailed in Table 3. Sexual dimorphism is evident in this species, males being smaller (20.7±1.07 mm; 18.67–22.18 mm) than females (29±1.25 mm; 27.73–30.79 mm). Juveniles and females commonly (~70%) shown dark grey flecks on dorsum.

**Coloration in life** (Figs 8 and 9): Body coloration is darkest in specimens observed by day, and brightest in specimens observed by night. By night, dorsum and flanks are bright greenish yellow; with or without black dots or orange blotches. Interobital bar, subocular stripes, scapular and sacral marks absent; a well-defined yellow line is present along the border or upper eyelid; sides of head colored as dorsum, black canthal stripe continuing to the mid-flank; groin and anterior surfaces of thighs are greenish yellow; posterior surfaces of thighs uniform is yellowish cream; ventral surfaces of belly are yellowish cream; throat, foot and hand are greenish tan. Dorsal surfaces of pads on Fingers II and III are pale yellow; iris coppery red stippled with gold and narrow black reticulations. By day, all bright surfaces on head, throat, flanks and limbs turn into dark greenish brown.

**Coloration in preservative:** Dorsum cream or tan, with or without brown flecks. Sides of head greyish brown; black canthal stripe well-defined. Forearms and hind limbs with or without dark brown marks. Venter, throat, chest, ventral surfaces of limbs, and palms cream; posterior surfaces of tarsus and plantar surfaces uniform cream. Darker coloration in preserved specimens might depend of the time in which each specimen was prepared as a voucher.

**Etymology:** Named for the Reserva Biológica Limoncocha, located in northern Amazonia of Ecuador. This area harbors the last remnant of natural forest in the Limoncocha Lagoon. Conservation programs are currently being developed together with local communities and Ecuadorian governmental entities such as the Ministerio del Ambiente del Ecuador.

**Natural history and distribution:** *Pristimantis limoncochensis* is known from 27 localities along the Amazonian evergreen lowland forest of southern Ecuador in Napo, Sucumbíos, and Orellana Provinces, and two localities from Putumayo and Caquetá Departments in Colombia, up to 593 m elevation. The area of occurrence is estimated to be about 28,139 km^2^ in lowland evergreen forest in eastern Ecuador and southern Colombia, but a wider distribution is likely, up to 177,042 km^2^ in the upper Amazon Basin (Fig 4). According to field notes and database records, specimens of *P*. *limoncochensis* were found active at night on leaves of low vegetation, up to 3 m above ground. It is suspected that is an inhabitant on forest canopy. Duellman [20], under the name of *Eleutherodactylus acuminatus*, describe the mating call like a “short, high whistle repeated infrequently”.

**Remarks:** This species was previously identified as *Eleutherodactylus acuminatus* by Duellman [20] and Lynch [45] for populations in the northern Amazon Basin of Ecuador. Recently, Beirne and Whitworth [92] report it for Yachana Reserve, in Napo Province. According with phylogenetic analyses, *P*. *limoncochensis* is sister to *P*. *omeviridis*.

#### *Pristimantis omeviridis* sp. nov. Ortega-Andrade, Rojas-Soto, Valencia, Espinosa de los Monteros, Morrone, Ron, and Cannatella

urn:lsid:zoobank.org:act:368B3560-8C0D-4B10-9CE6-E3EF0E1DFF7A

Figs 7–9.

**Holotype:** QCAZ 55392, an adult female collected on 8 March 2013 at Tambococha, N°0.97839, W°75.4256, 177 m elevation, by Fernando Ayala-Varela, Edwin Carrillo, Jorge Brito, Andrea Varela, Diego Quirola, Andrés Mármol and Javier Pinto, Orellana Province, Ecuador.

**Paratopotype:** QCAZ 55391 collected in amplexus with the holotype

**Paratypes:** Eight adult males (QCAZ 25296, 9215, 29685, 10215, 16748, 6832, 8281, GGU-IIAP 1031), five gravid females (QCAZ 18804, 17338, 10564, 8887, 31444), two sub-adult females (QCAZ 17866, 20403), two juvenile females (QCAZ 2564, FHGO 7192) and three juvenile males (QCAZ 17510, 17367, 55391), all collected from four localities in the northern Amazonian lowlands of Ecuador and Peru. ECUADOR: Napo Province: QCAZ 25296 collected on 4 October 2003 at Inner Vision Lodge, Río Arajuno, S°1.1018, W°77.5932, 370 m, by Kathryn Elmer and Tomi Sugahara; QCAZ 2564 on 24 May 1991 at Jatun Sacha Biological Reserve, 22.5 km road to Ahuano, S°1.0566, W°77.6161, 382m; QCAZ 9215 on 27 June 1987 at S side of the Río Napo: 6.5 km ESE Puerto Misahuallí, at La Cruz Blanca on Jatun Sacha Biological Reserve, S°1.05, W°77.6, 372m. Orellana Province: QCAZ 29685 on 16 February 2000 at Estación Científica Yasuní, S°0.6785, W°76.3963, 246m; FHGO 7192 on 14 October 2009 at Edén, S°0.5569, W°76.0891, 227 m, by Jorge Valencia; QCAZ 10215 on 20 July 1996 at Estación Biológica Tiputini, S°0.6388, W°76.1493, 219 m, by David Romo; QCAZ 17510 on 23 September 2000 at Estación Científica Yasuní, S°0.6772, W°76.4012, 249 m, by Zornitza Aguilar; QCAZ 8887 on 16 February 1996 at Estación Científica Yasuní, S°0.6772, W°76.4012, 249 m, by Fernando Nogales; QCAZ 17866 on 18 November 2001 at Estación Científica Yasuní km 7 1/2 Poza 1, S°0.9929, W°76.2037, 250m; QCAZ 16748 on 7 August 2001 at Estación Científica Yasuní Laguna 2, S°0.6713, W°76.4005, 241m; QCAZ 18804 on 14 January 2002 at Estación Científica Yasuní, S°0.6744, W°76.3971, 220 m, by Milton Zambrano; QCAZ 20403 on 12 February 2000 at Estación de Biodiversidad Tiputini, S°0.633, W°76.1473, 240 m, by Marcelo Díaz Proaño; QCAZ 6832 on 24 November 1994 at km 107 MAXUS road, S°0.9812, W°76.2476, 245 m, by Morley Read; QCAZ 17338 on at Parque Nacional Yasuní, S°0.99, W°76.25, 270m; QCAZ 8281 at Parque Nacional Yasuní, road Pompeya Sur–Iro, S°1.00, W°76.19, 244m; QCAZ 10564 on 2 February 1997 at Parque Nacional Yasuní, Estación Científica Yasuní (PUCE), km 6 road to Station, S°0.6796, W°76.4055, 249 m, by Juan M. Guayasamin and Xavier Cisneros; QCAZ 17367 on 25 February 1994 at Parque Nacional Yasuní, km 38 vía Pompeya—Iro, S°0.6536, W°76.4536, 236 m, by Stella de la Torre-Salvador and Santiago R. Ron; QCAZ 31444 on 26 February 2006 at Parque Nacional Yasuní, km 38 vía Pompeya—Iro, S°0.6536, W°76.4536, 236 m, by David Salazar and Erika Carrera. PERU: Loreto Department: GGU 1031 collected on 10 August 2008 at Curaray, Arabella, Lote 39, S°2.1483, W°75.0092, 154 m, by Giussepe Gagliardi.

**Characteristics:** A member of the *Pristimantis acuminatus* complex characterized by: (1) skin of dorsum smooth; dorsolateral folds absent; skin of belly coarsely areolate; discoidal fold barely evident; (2) tympanic annulus and membrane visible; (3) snout long, acuminate in dorsal view in females, sub-acuminate in males and juveniles; truncated and posteriorly inclined in profile; lips not flared, *canthus rostralis* angular in dorsal and lateral view, loreal region concave; (4) upper eyelid about 65% of inter-orbital distance, lacking tubercles; (5) dentigerous processes of vomer small, transverse, bearing 5–8 teeth; (6) males lacking vocal slits, vocal sac and nuptial excrescences; (7) fingers large and slender, first shorter than second; discs on outer fingers expanded, bluntly rounded, about 1.5x the width of digit proximal to pad; supernumerary tubercles large, rounded; (8) fingers bearing lateral fringes; (9) forearm bearing 2–4 ulnar tubercles, large; (10) heel lacking tubercles; outer border of tarsus smooth; inner border of tarsus bearing 3–4 tubercles or large tarsal fold; (11) two metatarsal tubercles; inner elliptical, about 2x the outer tubercle; supernumerary plantar tubercles small; (12) toes with lateral fringes; webbing absent; discs equal in size to those on fingers; Toe V longer than Toe III; (13) in life, anterior dorsum tends to be brighter greenish yellow than posterior portion and limbs, with or without scattered black, orange or brown blotches; groin and anterior surfaces of thighs uniformly pale yellow; belly and throat pale cream to white; ventral surfaces of hands and feet tan; black canthal stripe continuing posterior to the eyes until reaching the mid-flank; iris bronze, finely reticulated with black. In preservative, all yellow or green areas turn into cream or tan, anterior and posterior surfaces of thighs uniformly tan; venter cream; black canthal stripe, brown snout; (14) SVL in adult males 20.8±1.77mm (17.77–23.31); females with 28.6±1.92mm (26.1–30.91).

**Diagnosis:** Among greenish Amazonian *Pristimantis*, *P*. *omeviridis* sp. nov. is most similar to *P*. *enigmaticus* sp. nov., *P*. *pseudoacuminatus*, and *P*. *olivaceus* by having a differentiated tympanic annulus and membrane on skin. *Pristimantis omeviridis* is similar to *P*. *enigmaticus* in coloration and snout shape, but the *P*. *omeviridis* differs by smooth dorsum (shagreened in *P*. *enigmaticus*), having discoidal fold (absent), bearing large ulnar (small) and tarsal tubercles (small tarsal fold). *Pristimantis omeviridis* is like *P*. *pseudoacuminatus* in having a coarsely areolate belly, but the former species can be differentiated by bearing ulnar tubercles (absent), having smooth dorsum (with warts or conical tubercles), males lacking vocal slits (present) and by uniform color below eyes (with vertical brown bars), and larger body size (female SVL = 20.05−21.2 mm, male SVL = 13.7−16.2 mm). *Pristimantis omeviridis* can be distinguished from *P*. *olivaceus* by having smooth dorsum (shagreened), males lacking nuptial pads and vocal slits (present), enlarged ulnar and tarsal tubercles (small) and bright greenish yellow body (olive green with dark spots).

**Description of the holotype:** Body slender; head wider than body; slightly longer than wide, about 30% of SVL; snout long, acuminate in dorsal view, truncated and posteriorly inclined in profile; distance from nostril to corner of eye equal in length than diameter of eye; *canthus rostralis* straight in dorsal view, angular in cross section, sloping gradually to lips; lips not flared; internarial area not depressed, nostrils not protuberant, directed anterolaterally, situated about three-quarters the distance from the eyes to the tip of the snout; interorbital area flat, IOD 40% of head width; eye large, protuberant, its diameter about 3x depth of lip below eye and about 30% of head length; upper eyelid about 65% of inter-orbital distance, lacking tubercles; no interocular fold; cranial crests absent. Tympanic membrane and annulus prominent, round in shape, with supratympanic fold partially obscuring upper and posterodorsal edges; horizontal diameter of tympanum 30% of eye diameter, separated from eye by a distance of one third-tympanum length; postrictal tubercles fused to form a barely visible short ridge extending ventrolaterally from the tympanum; choana small, rounded, not concealed by the palatal shelf of maxillary arc; dentigerous processes transverse, angled postero-medially and closely separated; bearing 8 small teeth, visible; tongue elliptical, posterior border notched, not adherent to the floor of the mouth for about 40% of its length.

Skin on dorsum shagreened; no occipital ridges or dorsolateral folds; skin on flanks gradually shagreened to areolate; ventral surfaces of belly coarsely areolate; skin on ventral surfaces of chest, throat and thighs, smooth; discoidal folds barely visible; no thoracic fold. Forearm slender; fingers large and slender, all with oval (broader than long) pads, Fingers III-IV with large pads, all fingers with large discs; pad of Finger III about 1.5x wider than narrowest portion of penultimate phalanx; disc of Finger I distinctive smaller than those on other fingers; relative length of Fingers I<II<IV<III; subarticular tubercles large, subconical; supernumerary tubercles prominent, subconical; palmar tubercle bifid, 2x size of oval thenar tubercle; antebrachial tubercle small; three ulnar tubercles are present along inner edge of forearm, conical; outer edge of forearm shagreened, tubercles absent;

Hind limbs slender; tibia length about 50% of SVL; knee and heel lacking tubercles; foot length about 67% of SVL; outer border of tarsus smooth; inner border of tarsus bearing four tubercles, small; inner metatarsal tubercle oval, 3x size of round outer tubercle; supernumerary tubercles round, small; subarticular tubercles subconical, rounded; toes with non-crenulate lateral fringes; webbing absent between toes; pads of Toes III−V large, in all other pads and discs of toes like those of fingers; relative lengths I<II<III<V<IV; Toe III extending to one-half the distance between penultimate and ultimate subarticular tubercles on Toe IV; Toe V extending to distal edge of ultimate subarticular tubercle on Toe IV. Vent opening puckered, shagreen, not protruding, lacking tubercles on its border, located at upper level of thighs.

**Measurements (in mm) of the holotype:** Specimen QCAZ 37277 is an adult male with the following measurements: SVL = 30.3; HL = 10.1; HW = 9.3; ED = 3.3; EN = 3.4; IOD = 3.7; EW = 2.4; TD = 1.1; FL = 14.4; TL = 14.6; FtL = 20.3; HdL = 8.6; F3 = 1.8; T4 = 1.8. Proportions: HL/SVL = 0.33; HW/HL = 0.92; FL/SVL = 0.48; TL/SVL = 0.48; FtL/SVL = 0.67; EN/HL = 0.34; ED/HL = 0.33; IOD/HW = 0.4; TD/ED = 0.33.

**Variation:** Measurements and proportions of the specimens examined are given in Table 3. Sexual dimorphism is evident in this species, males being smaller (20.8±1.77 mm, 17.77–23.31 mm) than females (28.6±1.92 mm, 26.1–30.91 mm). Juveniles and males commonly show faded interorbital bar and dark brown flecks on dorsum. Most specimens have a pointed snout tip, whereas some have subacuminate to truncated snouts (e.g. QCAZ 10564, 17866). Ulnar tubercles are prominent in living individuals, whereas they tend to be less evident in preserved specimens (e.g. QCAZ 10564).

**Coloration in life** (Figs 7 and 8): At night, dorsum and flanks bright greenish yellow, with or without black dots or orange blotches. Interobital bar barely visible in males and juveniles; subocular stripes, scapular and sacral marks absent; a well-defined yellow line present along the border or upper eyelid; sides of head darker colored than dorsum, black canthal stripe continuing until reaching the mid-flank; groin and anterior surfaces of thighs pale yellow; posterior surfaces of thighs uniform yellowish cream; belly and throat pale yellow to white; ventral surfaces of hands and feet tan. Dorsal surfaces of pads on Fingers II and III greenish yellow; iris bronze with narrow black reticulations. At day, all bright surfaces on head, throat, flanks and limbs turn into dark greenish brown.

**Coloration in preservative:** Dorsum cream to tan, with or without brown flecks. Sides of head greyish brown, with a well-defined black canthal stripe. Forearms and hind limbs with or without dark brown marks. Venter, throat, chest, ventral surfaces of limbs, and palms cream; posterior surfaces of tarsus and plantar surfaces uniform cream. Darker coloration in preserved specimens might depend of the time in which each specimen was prepared as a voucher.

**Etymology:** The new species is named in honor of the Huaorani indigenous nation (including non-contacted Tagaeri and Taromenane people), and recognizes the jungle as essential for their physical and cultural survival in northern Amazonia of Ecuador. The specific name is derived from *ömë*, meaning “forest” or “jungle” in *Huao terero*, the Huaorani language, and the Greek *viridis*, meaning "green" in allusion to the green forest which is home to this group of mostly green species.

**Natural history and distribution:** *Pristimantis omeviridis* is known from 23 localities in the Amazonian evergreen lowland forest of northern Ecuador in Napo and Orellana Provinces, and one locality from Loreto Department in Peru, up to 382 m elevation. The area of occurrence is calculated to be about 22,950 km^2^ in lowland evergreen forest in eastern Ecuador and southern Colombia, but a wider distribution is likely, up to 147,787 km^2^ in the upper Amazon Basin (Fig 4). Specimens of *P*. *omeviridis* were found active at night on leaves of low vegetation, up to 3 m above ground, in primary and secondary forest. It is suspected that is an inhabitant of forest canopy.

## Discussion

*Pristimantis acuminatus* was considered a widely distributed nominal species in the upper Amazon Basin, but was suspected to be a complex of cryptic species [30]. This suspicion is corroborated by our study based on the review of its type series and the application of an integrative analyses (phylogenetic, morphological and ecological data) to a broad sample of specimens. As a result, we have described three new species and present additional information on the identity of *P*. *acuminatus*. We encourage examination of museum collections, accompanied by molecular analyses, and framing geographic distributions in an ecological context using ENMs are invaluable for the identification and validation of nominal species. In this context, following a first step reduction of taxon sampling is important to formulate a starting taxonomic framework. The protocol used herein has proven to be a very informative approach for evaluating the limits of cryptic species and could be applied to other species complexes (Fig 6).

### Integrative species delimitation

Delimitation of cryptic species within the *P*. *acuminatus* complex is supported by combined qualitative (morphological traits), quantitative (PCA-ordination and discriminant analyses on morphological and ecological space) and strongly supported molecular phylogenies. Although there are high levels of genetic diversity within Amazonian *Pristimantis* [37], the small genetic distances (1.6–3.8% for 16S) fall within typically used values species delimitation in other Neotropical frogs [93,94]. Nevertheless, genetic thresholds for setting species boundaries remain highly subjective; thus it is necessary to consider other sources of data to justify taxonomic action, including the description of new species [12,48,95].

Due the high diversity and variation within *Pristimantis* [19,29], several phylogenetic analyses have demonstrated lack of agreement with taxonomies based on morphology [11,37,96]. For example, Duellman and Lehr [30] firstly recognized *P*. *acuminatus* as a complex of species, because of the variation in tympanic condition within the nominal species. Here, we conclude that the condition of the tympanum is a useful character for diagnosing species within the *P*. *acuminatus* complex (Fig 10), but a combination of other traits such as skin texture, ulnar/tarsal tubercles and sexually dimorphic characters (vocal slits and nuptial pads in males), is preferable for confident delimitation of these species (Table 7).

Atympanic amphibians typically detect low-frequency sounds, in contrast to tympanic species, which process high-frequency sounds [97]. The absence of a tympanum in anurans from the tropical Andes seems to be relatively common, and has been suggested to be an adaptation to high-altitude habitats [98]. Nevertheless, nearly 25% of *Pristimantis* species in the Amazonian lowlands apparently lack a distinct tympanum (condition D sensu [30]) in Amazonian lowlands. The lack of a tympanum has been claimed to inhibit anuran vocalization [99], and nontympanic pathways of sound reception and ultrasonic signal systems may compensate for degeneration of the middle ear complex [100]. The evolutionary and functional implications of tympanum absence in frogs are intriguing due to the essential role in the middle ear in mating behavior, social communication, sexual selection and territoriality [101,102].

Multivariate analysis of quantitative data is useful when cryptic species cannot be identified based on qualitative morphological differences. PCA of morphometric traits indicated that snout-vent length and other traits related to limbs (foot, tibia, and femur length) and head (head length and inter-orbital distance) explain most of the variance. Based on phylogenetic analyses, we obtained a better discrimination of species limits by a consideration of sexual dimorphism (Fig 3). Consideration of morphometric variation among populations yielded successful discriminant classifications ranging from 81 to 93% (Table 4). Thus, statistical analyses enhance our diagnosis of species, even though occasional individuals are not correctly classified (e.g. juveniles, subadults).

The distribution of mitochondrial genetic variation within the *P*. *acuminatus* complex revealed a parallel phylogeographic history for monophyletic sister species (Fig 1). The partial overlap of distributions among northern and southern Amazonian populations suggests a history of parapatric divergence (clades A–B with respect to clades C–D) (Figs 1–5). The common ancestor of the complex split in northern and southern lineages approximately 6.2 Mya, at the end of late Miocene and early Pliocene [103]. During this timeframe, critical changes in climate, tectonics, river drainages, and biodiversity in concert with the major uplift of northern Andes, including extensive marine transgressions into the Amazon basin [26]. These events may have promoted biotic isolation in Amazonia, along with the effects of river barriers and vegetation changes during periods of aridity [104]. On the other hand, sister species demonstrate sympatry (between clades A–B and C–D), with the split from common ancestors during the late Pliocene (3.2 and 3.9 Mya, respectively). Accordingly, the diversification of sister species took place by isolation into the Napo Refugia [104], where environmental stability allowed extensive development of the Amazon terra firme forests [105].

In interpreting the ENMs, it is important to distinguish between the species’ known distribution, as indicated by the species records, the realized niche, and their potential distribution, the fundamental niche [71,106]. The former is that part of the fundamental niche in which a species has positive population growth rates, given the constraining effects of biological interactions and dispersal limitations (e.g. competition, habitat preferences). The fundamental niche refers to all the requirements for maintaining a positive population growth rate, disregarding biotic interactions [106]. We evaluated ENMs in the context of the fundamental niche, which identifies probabilistic suitability areas in geographic space (Fig 4). The extent of suitability areas varied among species, with a narrow ENM for *Pristimantis omeviridis* and a broad ENM for *P*. *enigmaticus* (Table 5). A reduction of about 75–86% in the extent of ENMs is observed when compared with occurrence areas as delimited by the convex hull polygon criterion (Table 5). Furthermore, distinctiveness among environmental niches is supported by PCA-env and discriminant analysis (Table 4, S8 Table).

The ENMs provides complementary information for species separation (Fig 6) in spite of their relatively high similarity (Table 6). The only exception is between *P*. *limoncochensis* from *P*. *omeviridis* and *P*. *enigmaticus*, in which the D value is not significantly different from random (Fig 4, Table 6). A debate on the concept of niche conservatism/divergence and its relation to ENM similarity indices has promoted hypotheses in an ecological-evolutionary framework [7,87,106]. From an evolutionary perspective, the close phylogenetic relationship between species of the *P*. *acuminatus* complex could explain the high similarity of the values calculated for ENMs (Table 6), suggesting niche conservatism (Table 6), suggesting niche conservatism [107]. However, a broader analysis including as larger sample size of related species of *Pristimantis* is needed to test this hypothesis.

The new species are suspected to be cryptic inhabitants of forest canopy, associated with bromeliads [44]. This bias in sampling and the resulting underestimation of the canopy fauna might explain the rarity of records in museum collections [108,109]. Despite the dearth of ecological data for *Pristimantis*, these frogs are thought to have small home ranges and low vagility [26,110], which is supported by genetic data in a very few species [111]. Local competition, source partition and microhabitat preferences can be leading ecological niche shift promoting speciation [112], in spite of the high values of environmental niche similitude found in this study [9,113].

A wider distribution is suspected for these species based on discovery of unknown populations in unexplored areas with a high probability of suitability. Examination of museum specimens reveals that several leaflitter dwellers, and canopy species were uncommon in herpetological collections [109,114,115]. New Amazonian species discovered and described in the past decade typically were based on few specimens [21,116,117], and the dearth of specimens was attributed mainly to sampling bias. This provides a challenge to biologists to critically evaluate the taxonomic status of widely distributed species, considering the growing evidence that an inventory of the amphibian fauna of Amazonia is far from complete, especially in complex and poorly surveyed microhabitats.

Despite the interest in developing methods of delimiting species, differences in methodologies, datasets and species recognition criteria often make species delimitation challenging in species-rich evolutionary radiations [1,12]. This is true for Amazonian *Pristimantis* frogs [11,37,118], where many cryptic species await to be described.

## Supporting Information

S1 Fig Phylogenetic trees of the *Pristimantis acuminatus* complex inferred from mitochondrial DNA. A) Bayesian tree inferred from 763 bp of 12S under a GTR+G model. B) Bayesian tree inferred from 564 bp of 16S under a GTR+G model. C) Bayesian tree inferred from 670 bp of COI under a HKY+I model. D) Maximum likelihood solution inferred by Poison tree processes (PTP) model on the best tree solution from GARLI. Values above branches are posterior probabilities and values below are non-parametric bootstrap proportions (values < 0.5 not shown).
(PDF)

S2 Fig Morphometric and environmental projections on principal components axes for the *Pristimantis acuminatus* complex. Specimens are projected among the three first principal component axes (PC1−3). Red dots correspond specimens analyzed from Clade A, green dots for Clade B, blue dots for Clade C and purple dots for Clade D, based on the phylogenetic analysis in Fig 1. SVL = Snout-Vent length.
(TIF)

S3 Fig Percentage of contribution of important bio-climate variables for ENMs. This result was obtained by the jackknife test on the 19 variables bioclimatic dataset as implemented in Maxent.
(TIFF)

S1 Table Specimens used in the phylogenetic analysis. Museum catalogue number, locality, sex, field collectors, date of collection, GenBank accession numbers [with acceptance] and sequences generated in this study for mtDNA 12S, 16S and Cytochrome Oxidase I are provided. PUCE = Pontificia Universidad Católica del Ecuador. Sex: M = male, F = female, GF = gravid female, J = juvenile, not sexed, JF = juvenile female, JM = juvenile male, "-" means the specimen was not sexed.
(XLSX)

S2 Table Specimens examined for environmental and ecological niche modeling analyses. "+" indicates a literature record from Duellman and Mendelson [21]. "*" means the specimen was photographed but not collected.
(XLSX)

S3 Table Primers employed in this study for PCR and DNA sequencing. (XLSX)

S4 Table Statistical support for partitioning schemes applied for phylogenetic analyses. Bayes factor comparison and statistical support for three proposed DNA sequence data partitioning schemes for phylogenetic analyses. Entries are twice the log of the Bayes factor in the comparison between models M0 and M1 [2ln(B_10_)]. Values in which 2ln(B_10_) >10 suggest very strong statistical support for more complex model.
(XLSX)

S5 Table Morphometric measurements used for principal component and discriminant analyses within *Pristimantis acuminatus* complex. Specimens were examined at Fundación Herpetológica Gustavo Orcés, Quito (FHGO); Museo de Zoología–Pontificia Universidad Católica del Ecuador, Quito (QCAZ); Centro de Ornitología y Biodiversidad, Lima (CORBIDI); collection of Giussepe Gagliardi at the Instituto de Investigaciones de la Amazonía Peruana, Iquitos (GGU-IIAP); Museum of Comparative Zoology, Harvard University (MCZ). Abbreviations used in Appendix are: M = male, F = female, GF = gravid female. Morphometric abbreviations are: SVL = snout–vent length; HL = head width; HW = head length; ED = horizontal eye diameter; IOD = Interorbital distance; EN = eye-nostril distance; EW = width of upper eyelid; TD = tympanum diameter (not used for PCA); FL = femur length; TL = tibia length; FtL = foot length; HdL = hand length; F3 = disc diameter on finger III; and T4 = disc diameter on toe IV. All measurements are in mm.
(XLSX)

S6 Table Principal component analysis on morphometric data for *Pristimantis acuminatus* complex. Loadings and percentage of explained variance for normed principal components I–IV based on morphometric variables. Bold numbers indicate highest loadings.
(XLSX)

S7 Table Discriminant analysis on morphometric data for *Pristimantis acuminatus* complex. Canonical scores, eigenvalues and percentage of variance explained for variables and Axes I–III of the discriminant analysis. Abbreviations are: SVL = snout–vent length; HL = head width; HW = head length; ED = horizontal eye diameter; IOD = Interorbital distance; EN = eye-nostril distance; EW = width of upper eyelid; FL = femur length; TL = tibia length; FtL = foot length; HdL = hand length; F3 = disc diameter on finger III; and T4 = disc diameter on toe IV. Bold numbers indicate highest absolute correlation between each variable and their respective discriminant function.
(XLSX)

S8 Table Principal components and discriminant analyses on environmental data for *Pristimantis acuminatus* complex. Loadings and percentage of variance explained for normed principal components (PCI–II) and discriminant axes (FI–III) based upon environmental variables used from WorldClim data set [73,119]. Bold numbers indicate highest loadings; asterisks indicate variables used to generate and validate ENMs.
(XLSX)
