# Supplementary figures and images for: Insights from Integrative Systematics Reveal Cryptic Diversity in Pristimantis Frogs (Anura: Craugastoridae) from the Upper Amazon Basin
Source: PLoS One. 2015 Nov 24;10(11):e0143392. doi: 10.1371/journal.pone.0143392 (PMC4658055; doi:10.1371/journal.pone.0143392)

a)

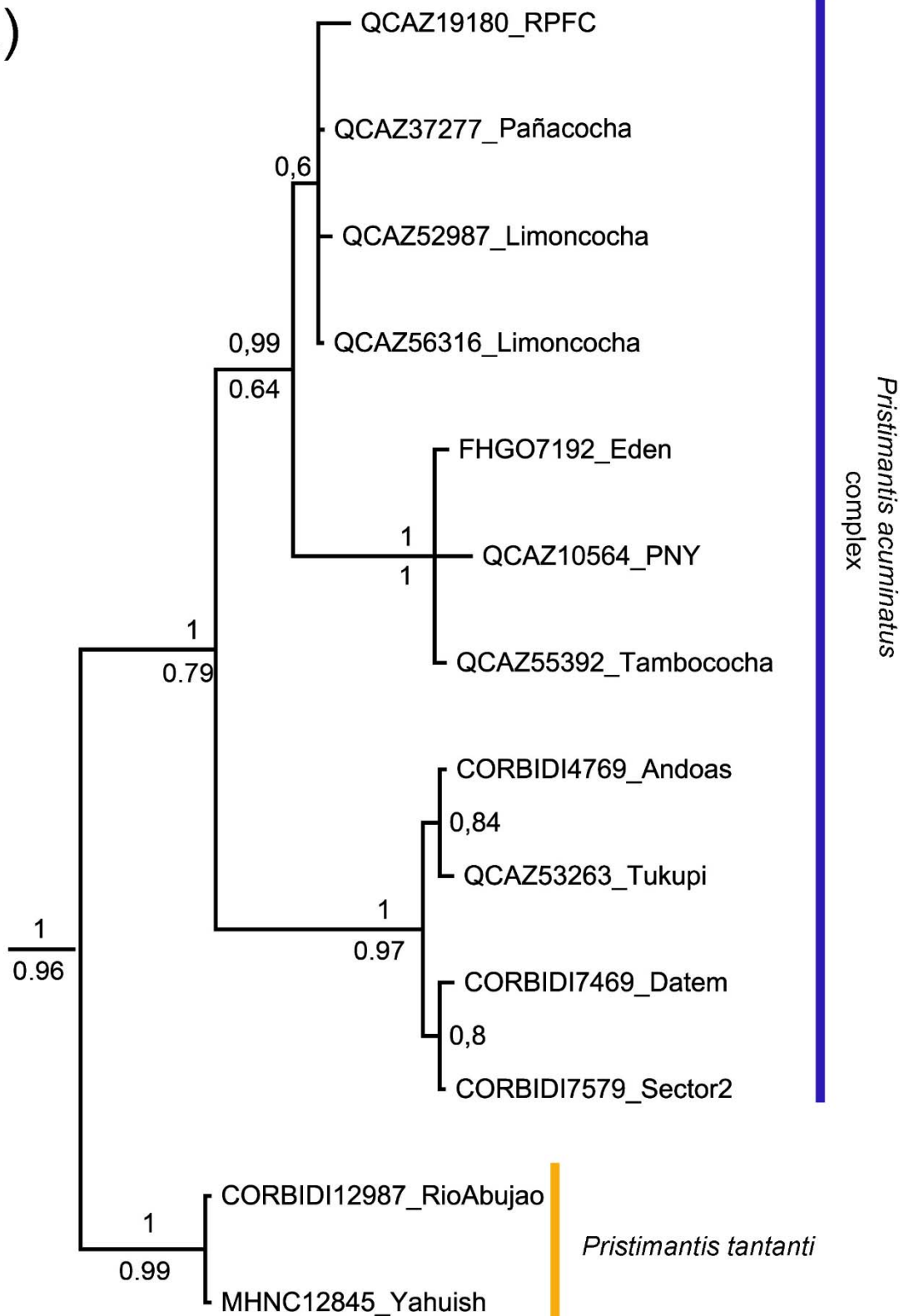

0.10 subs./site

b)

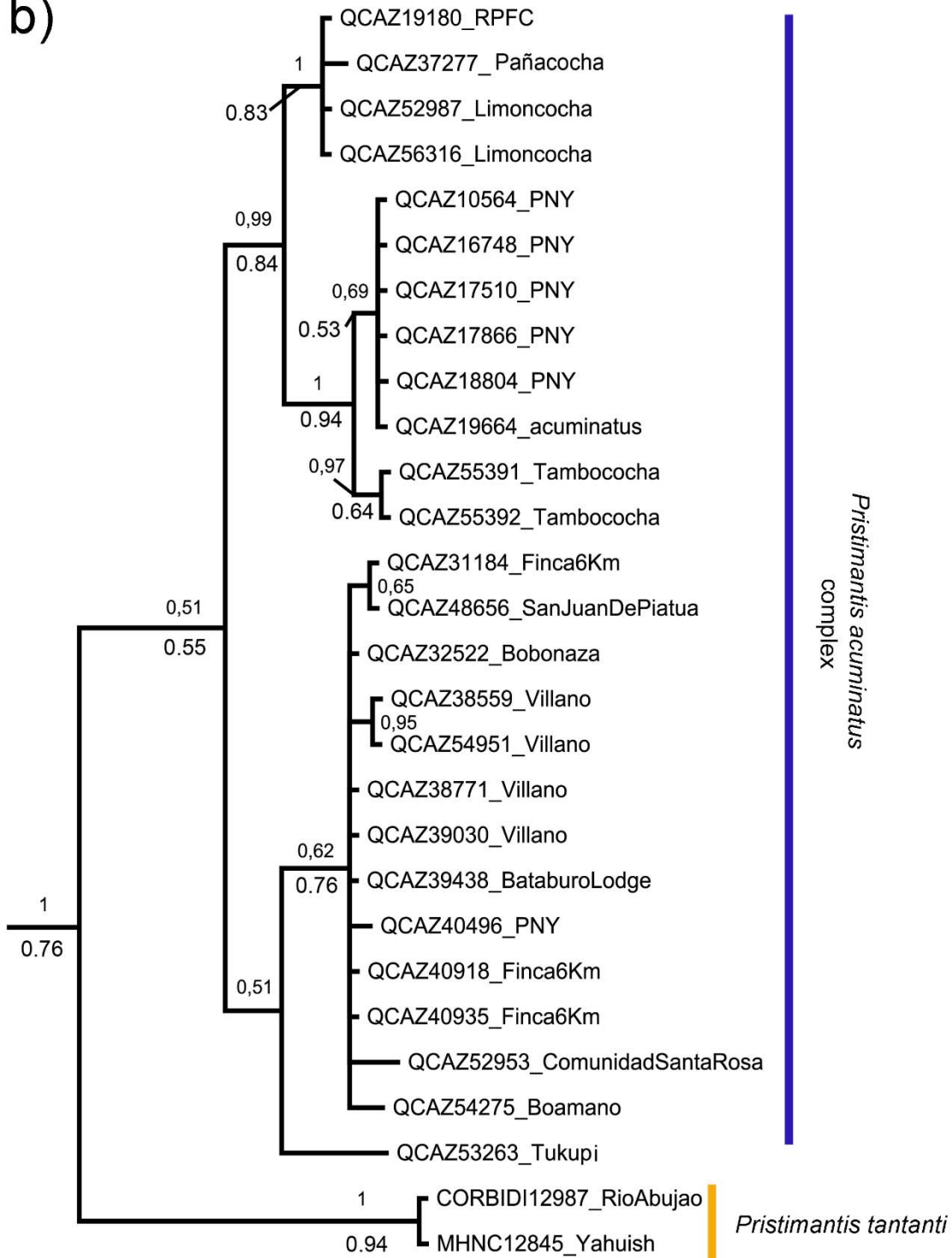

0.10 subs./site

c)

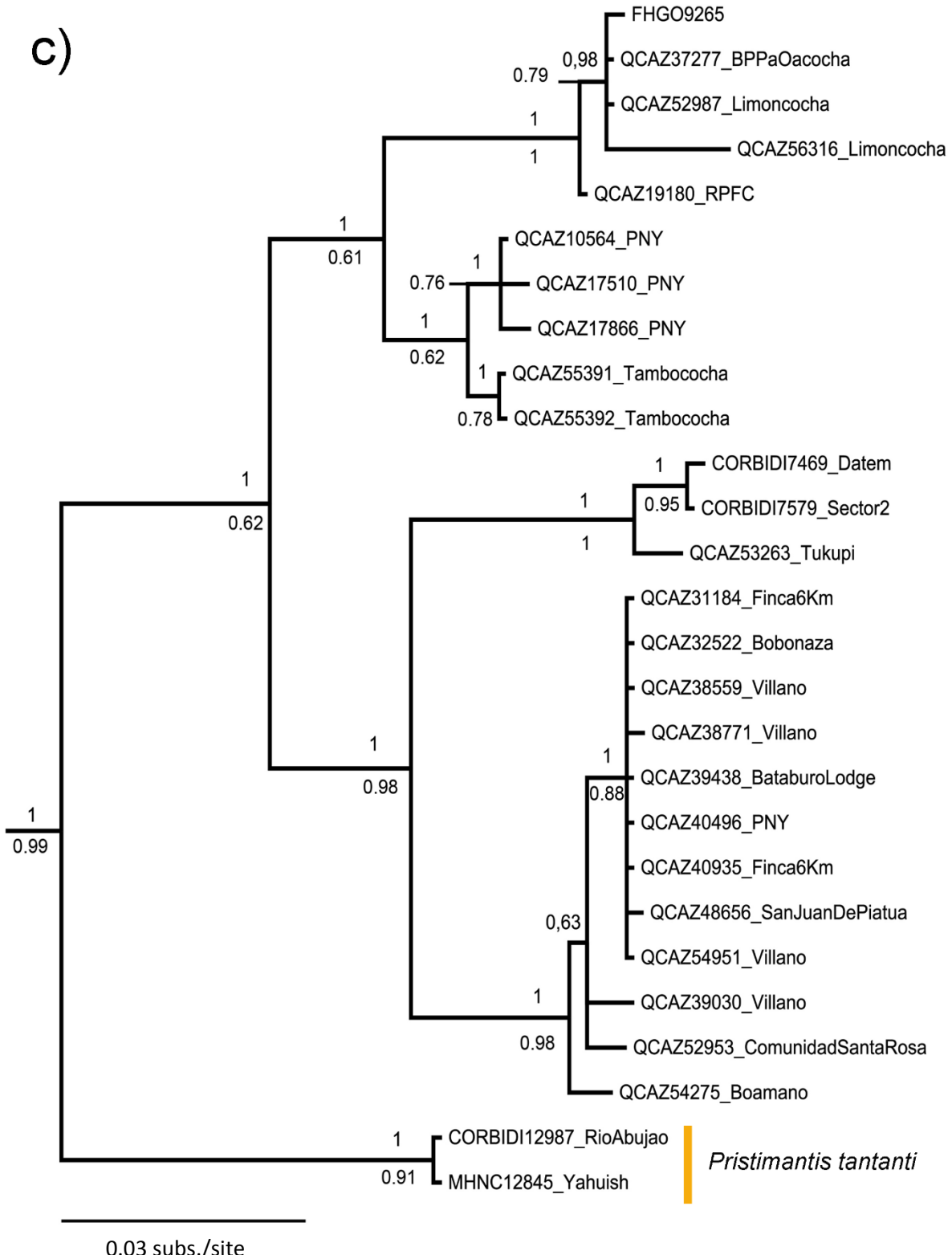

d)

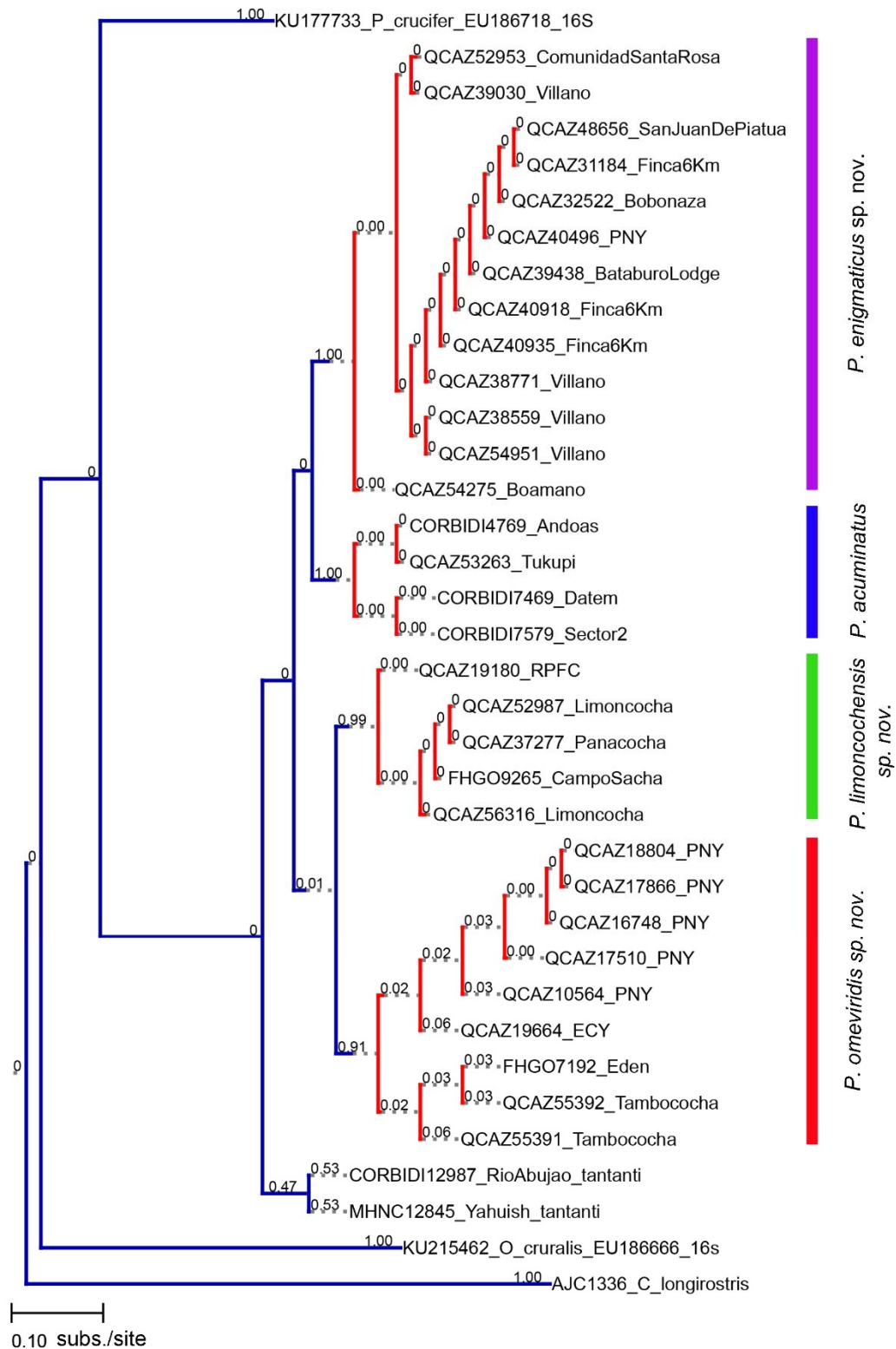

Supplement: S1 Fig — A) Bayesian tree inferred from 763 bp of 12S under a GTR+G model. B) Bayesian tree inferred from 564 bp of 16S under a GTR+G model. C) Bayesian tree inferred from 670 bp of COI under a HKY+I model. D) Maximum likelihood solution inferred by Poison tree processes (PTP) model on the best tree solution from GARLI. Values above branches are posterior probabilities and values below are non-parametric bootstrap proportions (values < 0.5 not shown). (PDF) [file pone.0143392.s001.pdf]

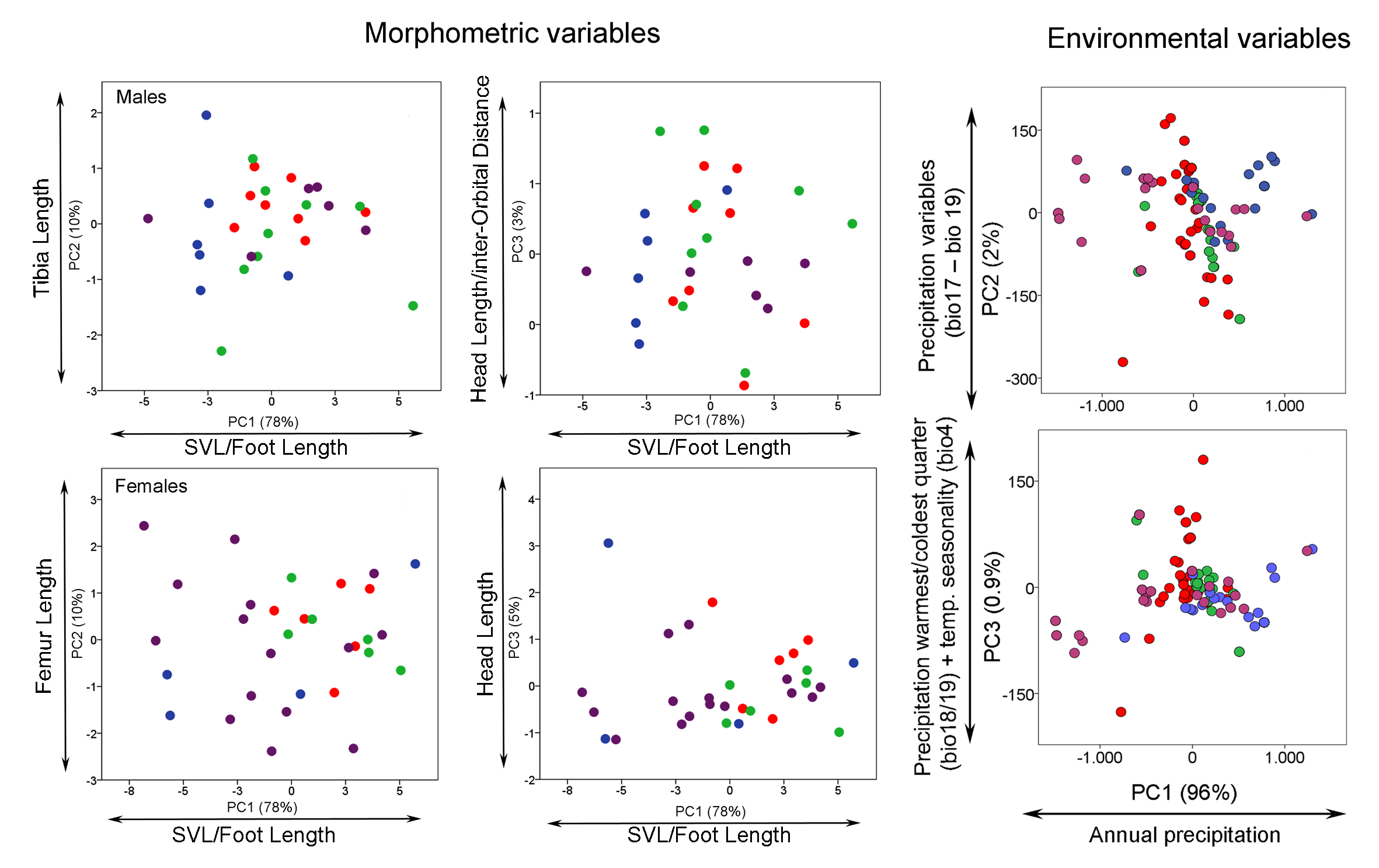

Supplement: S2 Fig — Specimens are projected among the three first principal component axes (PC1−3). Red dots correspond specimens analyzed from Clade A, green dots for Clade B, blue dots for Clade C and purple dots for Clade D, based on the phylogenetic analysis in Fig 1. SVL = Snout-Vent length. (TIF) [file pone.0143392.s002.tif]

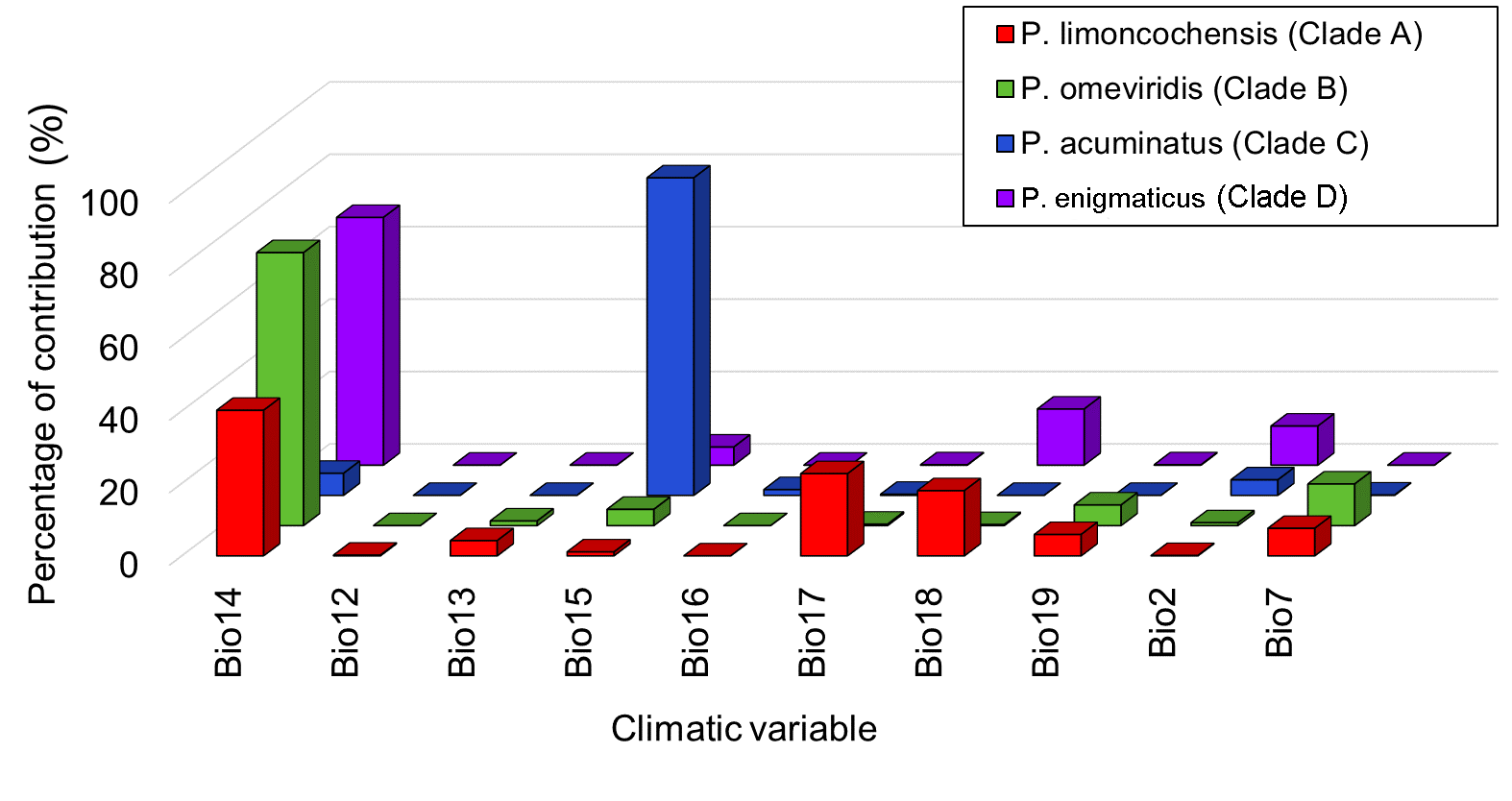

Supplement: S3 Fig — This result was obtained by the jackknife test on the 19 variables bioclimatic dataset as implemented in Maxent. (TIFF) [file pone.0143392.s003.tiff]
